# Entorhinal Silencing Reveals Energy Cascade Organization of Hippocampal Oscillations

**DOI:** 10.1002/hipo.70050

**Published:** 2025-12-08

**Authors:** Ben Zhao, Yu Qin, Sara N. Burke, Andrew P. Maurer

**Affiliations:** ^1^ Department of Neuroscience, McKnight Brain Institute, College of Medicine University of Florida Gainesville Florida USA; ^2^ Engineering School of Sustainable Infrastructure & Environment (ESSIE) University of Florida Gainesville Florida USA; ^3^ Department of Neuroscience University of Arizona Tucson Arizona USA; ^4^ Lead Contact

## Abstract

Hippocampal theta and gamma rhythms are often viewed as discrete channels supporting distinct cognitive operations. In particular, “gamma multiplexing” models propose that slow and fast gamma bands independently encode separate information streams or memory processes. Here, we present an alternative view: hippocampal oscillations form an interdependent process, governed by an energy cascade akin to turbulent flow across scales. In this framework, large‐amplitude, low‐frequency rhythms drive energy transfer to higher‐frequency oscillations, rather than acting as isolated carriers of segregated information. Using laminar local field potential recordings in freely moving mice, we show that optogenetic inactivation of the medial entorhinal cortex or CA1 reduces theta power, leading to proportional reductions across the entire gamma spectrum (60–100 Hz). These data support the perspective that the putative ‘slow gamma’ component (30–50 Hz) potentially reflects higher‐order theta harmonics rather than a distinct, independent rhythm. Moreover, locally generated gamma remains tightly coupled to theta, supporting an interdependent frequency spectrum modulated by network excitation. These findings challenge gamma multiplexing models and instead support an energy cascade framework, in which hippocampal gamma emerges from hierarchical, theta‐driven oscillatory dynamics. Recognizing gamma as part of an interdependent, turbulence‐like process reconciles contradictions in prior research and redefines how hippocampal oscillations contribute to cognition.

## 1 Introduction

The hippocampus and entorhinal cortex play crucial roles in learning and memory, spatial navigation, and other higher cognitive functions (O'Keefe and Nadel 1978; Squire 1992). Central to this coordination is the interplay between theta (4–12 Hz) and gamma oscillations (Vanderwolf 1969; Bragin et al. 1995; Buzsaki 2002). However, the fundamental nature of their rhythmic interaction, whether they operate as discrete, independent communication channels or as components of a single, interdependent process, remains a subject of intense debate.

Theta oscillations are closely linked to sensorimotor behaviors and involve both extrinsic inputs (e.g., from the medial septum and entorhinal cortex) and local circuit dynamics (e.g., Winson 1978; Vertes et al. 2001; Buzsaki 2002; Buzsáki 2005). Gamma oscillations, in contrast, emerge predominantly from local inhibitory‐excitatory interactions, with interneurons playing a crucial role in shaping pyramidal neuron firing at these faster timescales (Buzsáki and Chrobak 1995; Cobb et al. 1995; Wang and Buzsáki 1996; Olypher 1998; Buzsáki and Wang 2012). These rhythms interact, with gamma amplitude often modulated by theta phase, a phenomenon widely studied in theta–gamma coupling (Bragin et al. 1995; Chrobak and Buzsaki 1998; Chrobak et al. 2000; Schomburg et al. 2014).

In recent years, competing models have emerged to explain these oscillatory interactions. Gamma multiplexing and routing models posit that the gamma band is functionally segregated into distinct information streams: a slow gamma band (30–50 Hz) thought to relay input from CA3, and a fast gamma band (60–100 Hz) reflecting entorhinal input to CA1 (Colgin et al. 2009; Fernández‐Ruiz et al. 2017; Fernandez‐Ruiz et al. 2023). A recent revision to this theory suggests these streams originate from distinct entorhinal subdivisions, with the lateral entorhinal cortex driving slow gamma and the medial entorhinal cortex driving fast gamma (Fernández‐Ruiz et al. 2021). However, this revised model creates a potential anatomical paradox. Given that both lateral and medial entorhinal subdivisions are known to project to CA3 (Witter 2007), this would imply that CA3 is a site of mixed gamma convergence, rather than a purely segregated “slow gamma” generator. The model's plausibility is further challenged by a more fundamental line of evidence: the significant concern that “slow gamma” may not be a distinct oscillation, but rather a methodological artifact of non‐sinusoidal theta harmonics (Jones 2016; Scheffer‐Teixeira and Tort 2016; Zhou et al. 2019). Furthermore, dendritic low‐pass filtering properties (Golding et al. 2005; Vaidya and Johnston 2013) limit high‐frequency signal transmission from the dendrite to the axon hillock, challenging the feasibility of gamma‐based inter‐regional communication channels to control CA1 spike timing. Therefore, the multiplexing framework is challenged by both potential inconsistencies in its anatomical predictions, biophysical plausibility, and by evidence that one of its key components may be an artifact of the analysis itself.

Moreover, this framework has critical conceptual and empirical limitations. The brain's continuous, analog nature contrasts sharply with digital, discrete computational models (Maley 2011, 2020). The imposition of these rigid “communication channels” onto a continuous biological system is a prime example of the reductionist trap that Krakauer et al. (2017) caution against: conflating a simplified analytical algorithm with a complex neural implementation. This exact error was empirically demonstrated by Jonas and Kording (2017) in a microprocessor, where they showed that analyzing the system as a series of plausible “modules” failed to produce a meaningful understanding of how it actually works.

An alternative approach, rooted in statistical physics, views oscillations as an emergent energy cascade (Buzsaki and Draguhn 2004; Terrazas et al. 2005; Buzsaki 2006; Sheremet et al. 2016, 2018), where activity propagates across frequencies in a turbulence‐like manner rather than through discrete bands. Crucially, this does not imply uniform propagation between adjacent frequencies, but rather complex, nonlinear energy transfer that can skip scales or accumulate at specific resonant frequencies, as observed in physical turbulent systems (Frisch 1995). This principle of complex, scale‐dependent transfer is not merely a physical abstraction but has a direct neurobiological basis, as articulated by Buzsaki and Draguhn (2004):

“…*perturbations occurring at slow frequencies can cause a cascade of energy dissipation at higher frequencies* (Bak et al. 1987) *and that widespread slow oscillations modulate faster local events* (Steriade 2001; Csicsvari et al. 2003; Sirota et al. 2003). *These properties of neuronal oscillators are the result of the physical architecture of neuronal networks and the limited speed of neuronal communication due to axon conduction and synaptic delays* (Nunez 1995). *Because most neuronal connections are local* (Braitenberg and Schüz 2013), *the period of oscillation is constrained by the size of the neuronal pool engaged in a given cycle. Higher frequency oscillations are confined to a small neuronal space, whereas very large networks are recruited during slow oscillations* (Steriade 2001; Csicsvari et al. 2003). *These relations between anatomical architecture and oscillatory patterns allow brain operations to be carried out simultaneously at multiple temporal and spatial scales* (Buzsáki et al. 2004).” (Buzsaki and Draguhn 2004, 1926)

This description provides a clear physical mechanism for how a unified cascade can produce diverse spectral features: the anatomical scale of the recruited neural assembly directly constrains the frequency of the resulting oscillation.

This neurobiologically grounded view of an energy cascade has profound implications for interpreting oscillatory phenomena. A primary consequence, stemming from the local generation of high frequencies, is that these rhythms are ill‐suited for long‐distance information transfer. This perspective is strongly supported by recent findings in human systems neuroscience, where turbulence‐like dynamics and multi‐scale interactions consistent with energy cascades have been identified in both MEG and fMRI data (Deco and Kringelbach 2020; Deco et al. 2021). Ultimately, this framework recasts oscillations not as independent frequency bands encoding separate functions, but as emergent manifestations of a unified process of energy transfer across scales.

Supporting an integrated framework, Mizuseki et al. (2009) found that spiking between layer III of MEC and CA1, and between layer II of MEC and dentate gyrus–CA3, was offset by ~50–85 ms (~half a theta cycle), indicating that theta, rather than gamma, dominates inter‐regional coordination. More recently, Zhou et al. (2022) demonstrated that gamma coherence between MEC and CA1, once thought to reflect computational “routing,” instead arises from local network activity rather than direct gamma‐mediated cross‐regional communication.

These competing theoretical frameworks, therefore, generate distinct and falsifiable predictions that can be evaluated through circuit perturbations. The gamma multiplexing model predicts selective disruption: MEC inactivation should specifically reduce fast gamma (60–100 Hz) while preserving the 30–50 Hz ‘slow gamma’ band, with these channels operating independently across layers. The energy cascade model, in contrast, predicts interdependent disruption: circuit perturbations should produce proportional, broadband changes where theta power reductions drive corresponding gamma reductions across the entire spectrum.

The models also differ in their treatment of the 20–40 Hz band. Multiplexing/routing theories explicitly propose that this range potentially represents an independent ‘slow gamma’ oscillation. In contrast, extensive prior work has documented theta harmonics in this frequency range due to waveform asymmetries (Coenen 1975; Buzsaki et al. 1985; Sheremet et al. 2020). In direct contrast, a large and historically deep body of evidence suggests this same activity is not an independent oscillator at all, but a non‐linear harmonic of the fundamental theta rhythm, arising predictably from its non‐sinusoidal waveform (Scheffer‐Teixeira and Tort 2016; Zhou et al. 2019). Resolving this dispute is therefore not a minor issue; it is fundamental. Whether the 20–40 Hz band contains a distinct information channel or is an intrinsic, non‐linear feature of the primary theta rhythm represents an irreconcilable difference between the two models.

The present study, therefore, directly compares these competing frameworks, gamma multiplexing versus energy cascade models, to investigate the fundamental organization of hippocampal oscillations. We hypothesized that if gamma operates as an interdependent energy cascade, then inactivation of key inputs should produce broadband, proportional reductions in power across the spectrum, rather than the selective, channel‐specific disruptions predicted by multiplexing. To test this hypothesis and distinguish between the competing predictions, we performed a series of quantitative analyses, including (1) layer‐specific spectral analysis during circuit perturbations, (2) cross‐frequency power correlations to assess interdependence, (3) bicoherence analysis to distinguish harmonics from independent oscillations, and (4) coherence analysis across hippocampal laminae to evaluate the proposed routing mechanisms.

While Zutshi et al. (2022) demonstrated that MEC inactivation reduces both theta and gamma power in CA1, their study primarily focused on place cell dynamics and did not investigate whether these oscillatory reductions reflect interdependent energy transfer or independent channel disruption. Critically, they did not examine the quantitative mathematical relationships between frequency bands that would distinguish cascade from multiplexing mechanisms. Here, we extend their findings by directly testing these competing predictions through quantitative analysis of cross‐frequency relationships, phase coupling stability, and layer‐specific effects during circuit perturbations.

## 2 Materials and Methods

### 2.1 The Database

For the present study, we analyzed data from nine mice downloaded from the website of Buzsaki lab webshare (https://buzsakilab.nyumc.org/datasets/ZutshiI/Neuron2021). These data were generously provided by the Buzsáki laboratories (Zutshi et al. 2022). The mice used in this manuscript have been analyzed with different approaches. We were interested in the cross‐frequency coupling within the hippocampus CA1 layers during ipsilateral mEC or CA1 inactivation. To achieve mEC silencing, Zutshi et al. (2022) implanted a 200 μm optic fiber glued to a stainless‐steel ferrule to the shanks of the silicone probe. For local CA1 silencing, they attached a 100 μm optic fiber glued to a ceramic ferrule to the shanks of the silicon probe. The stimulation pulses were delivered optogenetically by a fiber coupled to a 450 nm blue laser diode at the central arm of the Figure 8 maze. We selected the following datasets: IZ18, IZ20, and IZ21 for the control, ipsilateral mEC, and CA1 inactivation; IZ24, IZ25 and IZ26 for the control condition; IZ27, IZ28, and IZ33 for the control, ipsilateral mEC, CA3, and ipsilateral mEC plus CA3 inactivation. Note, all inactivation was ipsilateral to the recording site.

### 2.2 Behavioral Paradigm

Animals were trained on a delayed spatial alternation task as described in Zutshi et al. (2022). The behavioral apparatus consisted of a Figure 8 maze with automated reward delivery and control systems. Animals performed 20 trials per session, with each session consisting of alternating blocks of stimulation and control conditions. During stimulation blocks, optogenetic inactivation was delivered for 1.5 s at the center arm of the maze during each of 10 consecutive trials, followed by 10 control trials without stimulation.

For the present analyses, data were collected from all segments of the maze environment, not restricted solely to the center arm. This approach differs from some analyses in Zutshi et al. (2022) that focused primarily on center arm activity (e.g., for phase locking and assembly analyses), allowing our study to provide a more comprehensive view of oscillatory dynamics across the entire behavioral environment. All analyses were restricted to periods when animals maintained mean running speeds above 16 cm/s to ensure consistent behavioral states and adequate theta power for spectral analyses.

### 2.3 The Time‐Series Analyses

To study the cross‐frequency coupling during different conditions, we first performed the current source density (CSD) analysis, and then the power spectral density (PSD) followed by the coherence, phase difference, cross‐correlation coefficient, and bicoherence. The CSD analysis is used to determine the layers of interest. They are pyramidal (sp), radiatum (sr), and lacunosum‐moleculare (slm) layer, respectively. Let $\Psi \left(z,t\right)$ denote a LFP sequence where z is the shank number and t the time. The CSD is typically approximated by the second order central difference of Ψ, which is

$$\frac{\partial^{2}\Psi \left(z,t\right)}{\partial z^{2}}=\frac{\Psi \left(z+h,t\right)-2\Psi \left(z,t\right)+\Psi \left(z-h,t\right)}{h^{2}},$$

where h is the inter‐electrode spacing, which can be 1 or the actual spacing value. This will result in N−2 CSD profiles, where N is the number of probes in a shank.

**FIGURE 1:**
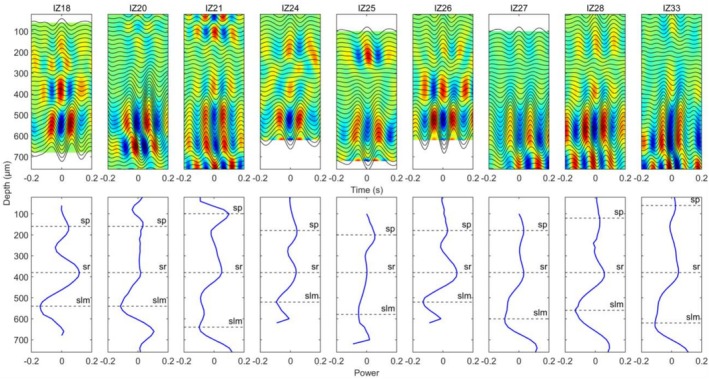
Top row: The CSD analysis to determine specific layers for different mice. The CSD was calculated on filtered theta traces (6–11 Hz) during awake behavior. Bottom row: The CSD amplitude at the 0‐time point. We identified the sp., sr, and slm layers. Each IZ number denotes a distinct mouse. All the subfigures were registered by the identified sr layers.

Usually, the data is prone to noise contamination. Differentiating data makes the result even more deteriorated. We applied a bandpass filter (6–11 Hz) to the wideband LFP signals to extract the theta waveforms and remove the fast‐changing noise, and then averaged the filtered LFP using the theta‐triggered peak to perform the CSD as a remedy measure (figure 1; for review, please see Chen et al. 2011).

The power spectral density (PSD) is obtained by the discrete Fourier transform (DFT). Let $x\left(n\right)$, n=0,1,2,…,N−1, be a sequence of N points, for example, a 1‐s recording of LFP. Such sequences are real. The transform $X\left(k\right)$, k=0,1,2,…,N−1 is defined by

$$X\left(k\right)=F\left(x\left(n\right)\right)=\sum_{n=0}^{N-1}x\left(n\right)\cdot e^{-i2\pi \frac{k}{N\;}n},$$

where F is the symbol of the Fourier transform. According to the Nyquist‐Shannon sampling theorem, the frequency range and corresponding indexing should be

$$f\left(k\right)=\frac{{\mathrm{kf}}_{s}}{N},k=0,1,2,\ldots ,\left\lfloor \frac{N}{2}\right\rfloor +1,$$

where $f_{s}$ is the sampling rate, and the amplitude of power is

$$G_{\mathrm{xx}}={\left|\frac{X\left(k\right)}{N}\right|}^{2},$$

which is the PSD of sequence $x\left(n\right)$. This PSD tells the power of a specific layer at different frequencies. Typically, the PSD is calculated using 1 or 2 s of LFP segment. These PSDs of LFP segments are averaged for a smooth representation, given that the studied object remains unchanged; for example, the running speed of corresponding segments should be within the designated intervals if the running speed is of interest.

For our analyses, we used 1‐s windows (1250 points at 1.25 kHz sampling rate) with 20% overlap (0.2 ratio). This window size was selected to optimize frequency resolution while maintaining adequate temporal precision for our research objectives. The choice of window size in spectral analysis involves a fundamental time‐frequency trade‐off: larger windows provide superior frequency resolution necessary for distinguishing theta harmonics from independent oscillations, while smaller windows can create spurious spectral features through windowing artifacts. Our 1‐s window provides 1 Hz frequency resolution, which is appropriate for identifying harmonic relationships and testing our theoretical predictions about energy cascade organization versus discrete oscillatory channels.

The coherence statistically measures the power transfer between two signals, meaning the similarity between them, assuming the system is linear. Let $x\left(n\right)$ and $y\left(n\right)$ be two sequences. The coherence is defined as

$$C_{\mathrm{xy}}\left(f\right)=\frac{{\left|G_{\mathrm{xy}}\left(f\right)\right|}^{2}}{G_{\mathrm{xx}}\left(f\right)G_{\mathrm{yy}}\left(f\right)},0\leq C_{\mathrm{xy}}\leq 1,$$

where $G_{\mathrm{xx}}\left(f\right)$ and $G_{\mathrm{yy}}\left(f\right)$ are the PSD as mentioned above, and $G_{\mathrm{xy}}\left(f\right)$ is the cross power spectral density (CPSD) between $x\left(n\right)$ and $y\left(n\right)$. The angle of the complex $G_{\mathrm{xy}}\left(f\right)$ is the phase difference between the two sequences. According to the Wiener–Khinchin theorem, $G_{\mathrm{xy}}\left(f\right)$ can be obtained by

$$G_{\mathrm{xy}}\left(f\right)=F\left(R_{\mathrm{xy}}\left(\tau \right)\right),$$

where $R_{\mathrm{xy}}\left(\tau \right)$ is the cross‐correlation function between $x\left(n\right)$ and $y\left(n\right)$. In the domain of digital signals, provided that $x\left(n\right)$ and $y\left(n\right)$ are ergodic. $R_{\mathrm{xy}}$ should be computed in a circular manner, which is

$$R_{\mathrm{xy}}\left(n\right)=x\left(n\right)*y\left(-n\right),n=0,1,2,\ldots ,N-1,$$

where * denotes the operator of convolution.

In order to quantify the precision of this estimate for a single animal (Figure 5), a 95% confidence interval was calculated for each spectrum using the Fisher z‐transform (as described for coherence in Bendat and Piersol 2011). This procedure first applies a variance‐stabilizing transformation to the coherence values:

$$z\left(f\right)=\text{atanh}\left(\sqrt{C_{\mathrm{xy}}\left(f\right)}\right)$$

The standard deviation of this transformed variable, $\sigma_{z}$, is dependent on the number of averaged segments, L:

$$\sigma_{z}=\frac{1}{\sqrt{2L}}$$

Confidence limits were then established in the z‐domain (as $z\left(f\right)\pm 1.96\cdot \sigma_{z}$) and subsequently transformed back to the original coherence scale to yield the final 95% confidence interval. This calculation was performed separately for each animal and experimental condition using the corresponding number of available segments, L.

The cross‐correlation coefficient marks the linear correlation between two sequences. In this work, the two sequences are the spectrograms of two specific layers, denoted by $G_{\mathrm{xx}}\left(f,t\right)$ and $G_{\mathrm{yy}}\left(f,t\right)$. The spectrogram records the PSD of a sequence at a specific time. In addition to f, $G_{\mathrm{xx}}$ and $G_{\mathrm{yy}}$ are also functions of t. For each frequency pair $\left(f_{1},f_{2}\right)$, the cross‐correlation coefficient accounts for the linearity between $G_{\mathrm{xx}}\left(f_{1},t\right)$ and $G_{\mathrm{yy}}\left(f_{2},t\right)$. This coefficient at $\left(f_{1},f_{2}\right)$ is given by

$$\rho_{X,Y}\left(f_{1},f_{2}\right)=\frac{\mathrm{cov}\left(G_{\mathrm{xx}}\left(f_{1},t\right),G_{\mathrm{yy}}\left(f_{2},t\right)\right)}{\sigma_{G_{\mathrm{xx}}\left(f_{1},t\right)}\sigma_{G_{\mathrm{yy}}\left(f_{2},t\right)}},-1\leq \rho_{X,Y}\left(f_{1},f_{2}\right)\leq 1,$$

where $\mathrm{cov}\left(G_{\mathrm{xx}}\left(f_{1},t\right),G_{\mathrm{yy}}\left(f_{2},t\right)\right)$ is the covariance, $\sigma_{G_{\mathrm{xx}}\left(f_{1},t\right)}$ is the standard deviation of $G_{\mathrm{xx}}\left(f_{1},t\right)$, and $\sigma_{G_{\mathrm{yy}}\left(f_{2},t\right)}$ is the standard deviation of $G_{\mathrm{yy}}\left(f_{2},t\right)$.

The covariance $\mathrm{cov}\left(G_{\mathrm{xx}}\left(f_{1},t\right),G_{\mathrm{yy}}\left(f_{2},t\right)\right)$ is

$$\begin{array}{c} \mathrm{cov}\left(G_{\mathrm{xx}}\left(f_{1},t\right),G_{\mathrm{yy}}\left(f_{2},t\right)\right)\\ \;=E\left(\left(G_{\mathrm{xx}}\left(f_{1},t\right)-\mu_{G_{\mathrm{xx}}\left(f_{1},t\right)}\right)\left(G_{\mathrm{yy}}\left(f_{2},t\right)-\mu_{G_{\mathrm{yy}}\left(f_{2},t\right)}\right)\right), \end{array}$$

where E is the expectation, $\mu_{G_{\mathrm{xx}}\left(f_{1},t\right)}$ is the mean of $G_{\mathrm{xx}}\left(f_{1},t\right)$, and $\mu_{G_{\mathrm{yy}}\left(f_{2},t\right)}$ is the mean of $G_{\mathrm{yy}}\left(f_{2},t\right)$. The matrix of cross‐correlation coefficients of $G_{\mathrm{xx}}\left(f_{1},t\right)$ and $G_{\mathrm{yy}}\left(f_{2},t\right)$ is not symmetric as $G_{\mathrm{xx}}\left(f_{1},t\right)$ and $G_{\mathrm{yy}}\left(f_{2},t\right)$ are spectrograms of two different layers, nor the diagonal entries are unity, which is unlike the symmetric auto‐correlation coefficient matrix. When $\rho_{X,Y}\left(f_{1},f_{2}\right)$ is positive, $G_{\mathrm{xx}}\left(f_{1},t\right)$ and $G_{\mathrm{yy}}\left(f_{2},t\right)$ are positively linearly correlated. When $\rho_{X,Y}\left(f_{1},f_{2}\right)$ is negative, $G_{\mathrm{xx}}\left(f_{1},t\right)$ and $G_{\mathrm{yy}}\left(f_{2},t\right)$ are negatively linearly correlated. When $\rho_{X,Y}\left(f_{1},f_{2}\right)$ is zero, $G_{\mathrm{xx}}\left(f_{1},t\right)$ and $G_{\mathrm{yy}}\left(f_{2},t\right)$ have no linear relationship.

Bicoherence is the normalized bispectrum. It enables the visualization of coupling of three frequency components. Let $f_{1}$ and $f_{2}$ denote two frequency components. The bicoherence checks the coupling of $f_{1}$, $f_{2}$, and $\left(f_{1}+f_{2}\right)$. There are several approaches to computing bicoherence. We chose the form proposed by Hagihira et al. (2001) and Hayashi et al. (2007), as it provides a solution to normalization. To obtain the bicoherence of a specific layer, suppose we have the LFP sequence and for each segment $x\left(n\right)$, the corresponding DFT is $X\left(k\right)$, or intuitively $X\left(f\right)$ by the above conversion. Assume there are total M segments. Let $X\left(f,t_{m}\right),m=1,2,3,\ldots ,M$ denote the DFT of different segment of $x\left(n\right)$, so $X\left(f,t_{m}\right)$ is also a function of time t. For each frequency pair of $\left(f_{1},f_{2}\right)$, perform the following operation

$$b\left(f_{1},f_{2}\right)=\frac{\left|\sum_{m=1}^{M}X\left(f_{1},t_{m}\right)X\left(f_{2},t_{m}\right)X^{*}\left(f_{1}+f_{2},t_{m}\right)\right|}{\sum_{m=1}^{M}\left|\;X\left(f_{1},t_{m}\right)X\left(f_{2},t_{m}\right)X^{*}\left(f_{1}+f_{2},t_{m}\right)\right|},0\leq b\left(f_{1},f_{2}\right)\leq 1,$$

where $b\left(f_{1},f_{2}\right)$ is the bicoherence, and * is the conjugate notation.

If $f_{1}$, $f_{2}$, and $\left(f_{1}+f_{2}\right)$ are perfectly coupled, for all $t_{m}$, the numerator is equal to the denominator, the bicoherence $b\left(f_{1},f_{2}\right)$ is one. That is total phase coupling. On the contrary, if $f_{1}$, $f_{2}$, and $\left(f_{1}+f_{2}\right)$ do not have phase coupling, the terms of the numerator cancel. $b\left(f_{1},f_{2}\right)$ becomes zero. That means these phases are random.

### 2.4 A Note on Terminology

We refer to the 20–40 Hz frequency range in terms of its harmonic relationship to theta, consistent with extensive historical precedent in the field (Harper 1971; Coenen 1975; Leung et al. 1982; Buzsaki et al. 1985; Ning and Bronzino 1989; Schomburg et al. 2014).

### 2.5 Statistical Analyses

Statistical analyses were performed using SPSS (v27) and Python (using statsmodels and scipy libraries). Our framework distinguished between a priori confirmatory analyses testing our core hypotheses and post hoc exploratory analyses suggested during review. For all tests, the significance threshold was set at *α* ≤ 0.05.

#### 2.5.1 Confirmatory (A Priori) Analyses

To test our primary hypotheses on the effects of MEC and CA1 inactivation, we used linear mixed‐effects models (LMMs) to accommodate the unbalanced experimental design resulting from different subsets of animals receiving various treatment combinations. In these models, condition (e.g., Control, Ipsi mEC, CA1) and recording layer (e.g., SP, SR, SLM) were included as fixed effects, with animal identity as a random effect to account for the repeated‐measures structure. Power values were log‐transformed to meet assumptions of normality.

To control for multiple comparisons across our planned analyses, we controlled the False Discovery Rate (FDR) using the Benjamini‐Hochberg method across conceptually related “families” of tests. For example, the 12 comparisons for oscillatory power (4 frequency bands × 3 layers) were treated as one family.

#### 2.5.2 Exploratory (Post Hoc) Analyses

A set of exploratory analyses was conducted at a reviewer's request to examine the effects of CA3 inactivation, alone and in combination with MEC inactivation (*n* = 3). Given that these analyses were not part of the original study design, they were treated as a distinct, post hoc conceptual family. To ensure statistical rigor, we applied the Benjamini‐Hochberg FDR correction to the *p*‐values generated within this specific set of comparisons. While these analyses are constrained by a small sample size, the reported FDR‐corrected *p*‐values provide a rigorous assessment of these exploratory findings.

#### 2.5.3 Effect Sizes and Power

Cohen's *d* was calculated for key comparisons and interpreted using established benchmarks: negligible (∣*d*∣ < 0.2), small (0.2 ≤ ∣*d*∣ < 0.5), medium (0.5 ≤ ∣*d*∣ < 0.8), large (0.8 ≤ ∣*d*∣ < 1.2), and very large (∣*d*∣ ≥ 1.2). Sample sizes for our primary confirmatory analyses (*n* = 6–9) provided ~80% power to detect effect sizes (Cohen's *d*) > 1.3.

## 3 Results

To arbitrate between the competing predictions of the cascade and multiplexing models, we performed a series of analyses designed to move from broad effects to specific mechanisms. We first quantified the effects of circuit perturbations on the overall power spectrum to test for selective versus interdependent disruption. We then examined the organization of these spectral components using cross‐frequency power correlations. Finally, to investigate the underlying mechanisms of coordination, we analyzed linear (coherence) and non‐linear (bicoherence) phase relationships across hippocampal layers.

### 3.1 Effects of Medial Entorhinal Cortical Versus CA1 Inactivation on CA1 Local Field Potential Organization

Current source density (CSD) analysis was used to identify the stratum pyramidale (sp), stratum radiatum (sr), and stratum lacunosum‐moleculare (slm) hippocampal layers across different mice (Figure 1). The CSD was calculated on filtered theta traces (6–11 Hz) during awake behavior. Because the different layers have distinct afferent input, with MEC perforant path fibers synapsing in the slm and CA3 Schaffer collaterals synapsing in the sr (Amaral and Witter 1995; van Strien et al. 2009), this layer‐specific approach is crucial for understanding the complex dynamics of hippocampal oscillations and their generation (Buzsaki et al. 1986; Bragin et al. 1995; Montgomery et al. 2009).

**FIGURE 2:**
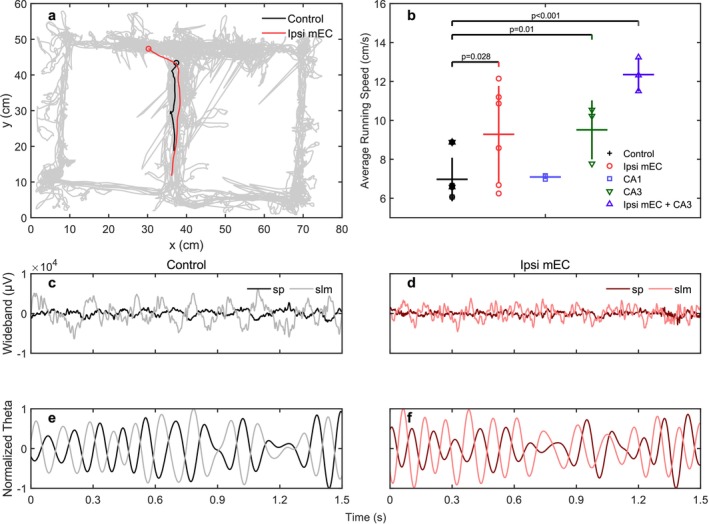
An example showing 1.5‐s traces for the control and ipsi mEC stimulation. (a) The trace in the maze during the 1.5 s for both conditions. The circle marks indicate the initial positions of the traces. The gray region depicts the overall trace of the whole session. This example is from IZ18. (b) The average running speed for all mice during all conditions. Each color mark denotes a mouse. The horizontal bars indicate the mean value. The vertical lines indicate 1 standard deviation. These legends apply to the following figures. The *p*‐values with significance between pairs are indicated in value. (c, d) The wideband LFP signals for the sp. and slm layers. When the ipsi mEC is inactivated, the amplitude is lower compared with the control condition. (e, f) The theta (6–11 Hz) phase difference between sp. and slm is generally stable in control, while during ipsi mEC, some theta cycles exhibit very different phase lag.

Figure 2a shows a representative running trace with the red segment indicating when 1.5 s of diode stimulation was delivered to optogenetically inactivate the MEC. To ensure that observed changes in oscillatory activity were not a secondary consequence of differences in locomotor behavior, we first analyzed average running speeds across all inactivation conditions (Figure 2b). A one‐way ANOVA revealed a significant effect of inactivation condition on running speed (*F*(4, 19) = 7.94, *p* = 0.0006). Notably, ipsilateral MEC inactivation significantly increased running speed compared to control conditions (Control: 6.97 ± 0.37 cm/s; Ipsi mEC: 9.28 ± 1.01 cm/s; 33.2% increase, *p* = 0.028, Cohen's *d* = 1.20). This hyperlocomotion was even more pronounced when MEC and CA3 were inactivated together (12.35 ± 0.50 cm/s; 77.2% increase, *p* < 0.001, *d* = 5.41). CA3 inactivation alone also increased running speed (9.51 ± 0.87 cm/s; 36.4% increase, *p* = 0.010, *d* = 1.91), while CA1 inactivation had no effect on locomotion (7.09 ± 0.06 cm/s; *p* = 0.863, *d* = 0.15; Table 1).

**TABLE 1:** Effect of inactivation condition on running speed.

Condition	N_Animals	Mean_cm/s	SEM	Vs_Control_p	Cohen_d	Percent_Change
Control	9	6.97	0.37	—	—	—
Ipsi_mEC	6	9.28	1.01	0.028	1.2	33.20%
CA1	3	7.09	0.06	0.863	0.15	1.70%
CA3	3	9.51	0.87	0.01	1.91	36.40%
mEC + CA3	3	12.35	0.5	< 0.001	5.41	77.20%

*Note:* MEC inactivation causes significant hyperlocomotion, with running speeds increasing 33%–77% depending on the extent of manipulation. This paradoxical increase in locomotion despite reduced theta power indicates that MEC inputs normally constrain movement speed while simultaneously organizing theta oscillations. The dissociation between increased speed and decreased theta power during MEC inactivation demonstrates that entorhinal inputs are essential for maintaining the normal relationship between locomotor behavior and hippocampal oscillations.

It was evident in the raw LFP traces that MEC inactivation simultaneously led to an overall reduction in LFP power (Figure 2c–f). Because faster running speeds are typically associated with greater theta power (e.g., Maurer et al. 2005; Terrazas et al. 2005; Sheremet et al. 2019; Qin et al. 2023), this paradoxical dissociation (where locomotion increases while oscillatory power decreases) indicates that entorhinal inputs are essential for maintaining the normal speed–theta relationship.

### 3.2 Overall Changes in Power by Condition

**FIGURE 3:**
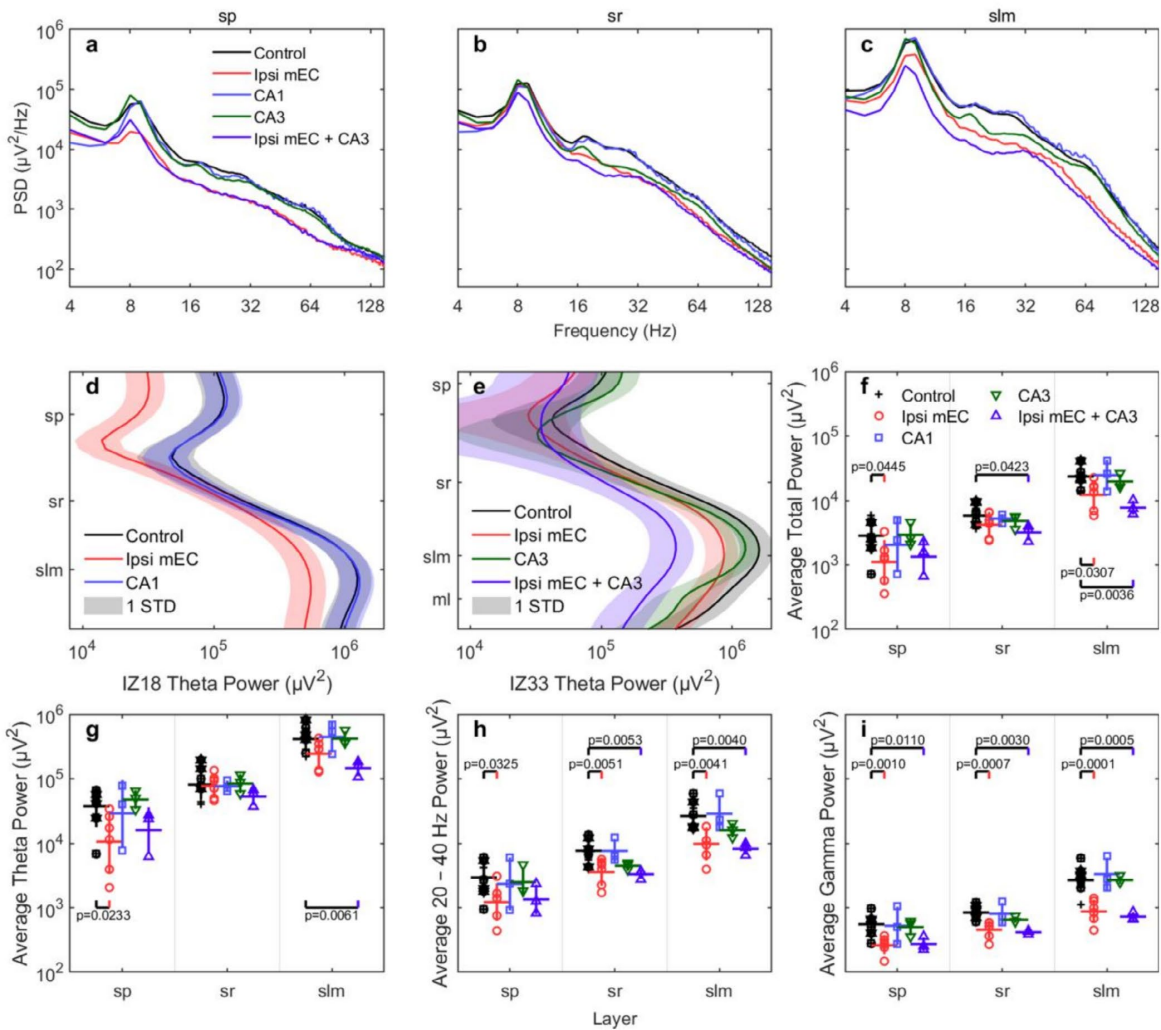
The average power spectral density (PSD) for running speed higher than 16 cm/s for the sp., sr, and slm layers and the control, ipsi mEC, CA1, CA3, and ipsi mEC + CA3 conditions. (a) The control, CA1, and CA3 inactivation do not share significant differences starting from the theta frequency in sp. When ipsi mEC is inactivated, huge power declines are observed, specifically in the theta, theta harmonics, and gamma ranges. The same pattern is observed for the ipsi mEC + CA3 inactivation. (b) The ipsi mEC inactivation does not change much of the power of theta in sr, while the CA3 inactivation decreases the power for frequencies higher than the base theta without disturbing the harmonic theta and potentially gamma. (c) The relationship in slm is similar to that in sp. except that the theta power percentage drop during ipsi mEC inactivation is smaller compared with the control and CA1 conditions. The ipsi mEC + CA3 inactivation suppresses the overall PSD collectively. These PSDs were averaged by 9 mice for the control, 6 mice for the ipsi mEC, 3 mice for the CA1, 3 mice for the CA3, and 3 mice for the ipsi mEC + CA3 inactivation. (d) The mean theta power is high at slm and low between sp. and sr. There is no significant difference between control and CA1 while the theta power drops during ipsi mEC. Such difference near sr is minimal. The animal is IZ18. (e) We observe a similar pattern for both the control and ipsi mEC conditions. The CA3 inactivation gradually decreases the theta power starting from the middle of the sp. and sr layers. With ipsi mEC + CA3 inactivation, a greater theta power decline is present particularly from sr to deeper layers. The animal is IZ33. (f–i) The average total power (4–150 Hz), theta power (7–10 Hz), theta harmonic power (20–40 Hz), and gamma power (60–100 Hz) are similar. The significance is marked by value between control and the other condition. Please see Figure S3 for a zoomed‐in depiction of the statistics subpanels.

Figure 3a–c shows the average power spectral density for different layers under different inactivation conditions. To examine how entorhinal inputs contribute to oscillatory power in CA1, we analyzed local field potential power across multiple frequency ranges using linear mixed‐effects models with condition and layer as fixed effects and animal as a random effect. This approach accommodated our unbalanced experimental design, where different subsets of animals received various treatment combinations. Based on our a priori hypothesis that MEC input drives CA1 oscillatory power, we designated Control versus Ipsi mEC comparisons as planned comparisons and performed 12 tests (4 frequency bands × 3 layers) with false discovery rate (FDR) correction using the Benjamini‐Hochberg method.

When total power (4–150 Hz) was analyzed, linear mixed‐effects modeling revealed a significant main effect of inactivation condition (*F*(4, 70) = 8.92, *p* < 0.001). Ipsilateral MEC inactivation produced layer‐specific reductions, with the largest effect in stratum lacunosum moleculare (44.6% reduction, p_FDR = 0.049, Cohen's *d* = −1.23) and a significant effect in stratum pyramidale (52.9% reduction, p_FDR = 0.059, *d* = −1.13), while stratum radiatum showed a non‐significant reduction. In contrast, neither CA1 nor CA3 inactivation alone significantly affected total power. An LMM analysis of the CA3 condition confirmed no significant change in any layer (SP: −14.8% change, *p* = 0.560, Cohen's *d* = −0.40; SR: −19.6% change, *p* = 0.316, *d* = −0.76; SLM: −11.9% change, *p* = 0.358, *d* = −0.68; *n* = 3). The combined ipsilateral MEC + CA3 inactivation produced the largest power reductions observed, with total power in stratum lacunosum moleculare reduced by 67.8% (*p* = 0.0036, uncorrected; *d* = −3.25), though these results should be interpreted cautiously given the small sample size (*n* = 3).

### 3.3 Band‐Specific Changes in Power by Condition

To better understand the nature of this broadband power reduction, we next quantified the impact of inactivation on specific hippocampal frequency bands: theta (7–10 Hz), the associated harmonics (20–40 Hz), and gamma (60–100 Hz; Figure 3g–i). Theta power (7–10 Hz) was significantly reduced in stratum pyramidale during MEC inactivation (Control: 46,472 ± 7360 μV^2^; Ipsi mEC: 17,104 ± 5786 μV^2^; 63.2% reduction, *p* = 0.023, p_FDR = 0.047, *d* = −1.29), with a large but non‐significant reduction in stratum lacunosum moleculare (39.8% reduction, *p* = 0.077, p_FDR = 0.092, *d* = −1.00). As previously reported, and evident in Figure 3d,e, theta power showed a significant laminar gradient, with power highest in the slm, intermediate in the sr, and lowest in the sp. This significant disruption of the primary, low‐frequency theta rhythm (the proposed driver of the cascade) provides the necessary foundation for examining the interdependent effects on higher frequencies. A LMM analysis of the CA3 inactivation confirmed no significant change in theta power, with negligible effect sizes in all layers (SP: −2.7% change, *p* = 0.787, Cohen's *d* = −0.18; SR: −0.5% change, *p* = 0.938, *d* = −0.05; SLM: −2.8% change, *p* = 0.841, *d* = −0.13; *n* = 3).

We next quantified the impact of inactivation on the 20–40 Hz harmonic range (Figure 3h). Harmonic power showed significant reductions across all layers during MEC inactivation, with the largest effect in stratum lacunosum moleculare (60.6% reduction, *p* = 0.004, p_FDR = 0.012, *d* = −1.76), followed by stratum radiatum (52.9% reduction, *p* = 0.005, p_FDR = 0.012, *d* = −1.72) and stratum pyramidale (54.3% reduction, *p* = 0.033, p_FDR = 0.049, *d* = −1.23; Table 2). In stark contrast, an LMM analysis of the CA3 inactivation confirmed no significant change in harmonic power in any layer (SP: −2.7% change, *p* = 0.865, Cohen's *d* = −0.11; SR: −11.9% change, *p* = 0.332, *d* = −0.73; SLM: −6.3% change, *p* = 0.384, *d* = −0.63; *n* = 3).

**TABLE 2:** Planned comparisons (control vs. Ipsi mEC) with FDR correction.

Frequency band	Layer	CON (Mean)	CON (95% CI)	Ipsi‐mEC inactivation (mean)	Ipsi‐mEC inactivation (95% CI)	Percent change	Magnitude change (95% CI)	P	P_FDR	Cohen's *d*	Effect size
Total (4–150 Hz)	SP	3547.1	(2228.9, 4865.3)	1670.9	(311.7, 3030.0)	52.90%	(−3562.7, −189.8)	0.045	0.0593	1.13	Large
Total (4–150 Hz)	SR	6139.9	(4415.6, 7864.3)	4557	(2608.2, 6505.7)	25.80%	(−3898.2, 732.3)	0.161	0.1755	0.77	Medium
Total (4–150 Hz)	SLM	27263.8	(18681.5, 35846.1)	15098.6	(7427.9, 22769.3)	44.60%	(−22470.9, −1859.4)	0.031	0.0488*	1.23	Very Large
Theta (7–10 Hz)	SP	46472.4	(29500.4, 63444.4)	17103.7	(2231.1, 31976.2)	63.20%	(−49593.7, −9143.9)	0.023	0.0467*	1.29	Very Large
Theta (7–10 Hz)	SR	85,372	(56851.1, 113892.9)	77392.7	(50607.1, 104178.3)	9.30%	(−42931.7, 26973.2)	0.777	0.7773	0.16	Negligible
Theta (7–10 Hz)	SLM	485774.2	(310998.4, 660550.0)	292320.6	(150474.5, 434166.7)	39.80%	(−396117.3, 9210.2)	0.077	0.0923	1	Large
Harmonics (20–40 Hz)	SP	3781.5	(2401.2, 5161.7)	1729.4	(551.3, 2907.6)	54.30%	(−3680.8, −423.2)	0.033	0.0488*	1.23	Very Large
Harmonics (20–40 Hz)	SR	8547.7	(6026.5, 11068.9)	4030	(2242.5, 5817.4)	52.90%	(−7328.2, −1707.3)	0.005	0.0123*	1.72	Very Large
Harmonics (20–40 Hz)	SLM	32113.5	(20921.9, 43305.0)	12663.9	(5932.9, 19394.9)	60.60%	(−31493.9, −7405.2)	0.004	0.0123*	1.76	Very Large
Gamma (60–100 Hz)	SP	612.3	(472.6, 751.9)	288	(200.0, 376.0)	53.00%	(−475.8, −172.6)	0.001	0.0039*	2.23	Very Large
Gamma (60–100 Hz)	SR	851.2	(683.4, 1018.9)	459.1	(336.3, 581.9)	46.10%	(−580.7, −203.5)	0.001	0.0039*	2.28	Very Large
Gamma (60–100 Hz)	SLM	2915.8	(1886.4, 3945.3)	843.3	(553.7, 1132.9)	71.10%	(−3114.2, −1030.9)	0.001	0.0013**	2.95	Very Large

*Note:* Effects of ipsilateral medial entorhinal cortex inactivation on CA1 oscillatory power across frequency bands and layers. Power spectral density values (mean ± SEM in μV^2^) for Control and Ipsi mEC conditions across four frequency bands and three hippocampal layers. The 95% confidence intervals are shown for both group means and the difference between conditions. Planned comparisons (*n* = 12) were performed using independent *t*‐tests on log‐transformed data with false discovery rate (FDR) correction using the Benjamini‐Hochberg procedure. Cohen's *d* effect sizes were calculated on the log‐transformed scale and interpreted following standard guidelines: negligible (|*d*| < 0.2), small (0.2 ≤ |*d*| < 0.5), medium (0.5 ≤ |*d*| < 0.8), large (0.8 ≤ |*d*| < 1.2), and very large (|*d*| ≥ 1.2). Asterisks indicate comparisons that survived FDR correction (*q* < 0.05). Double asterisks indicate the comparison that additionally survived Bonferroni correction for all 120 comparisons performed (*p* < 0.0004). Sample sizes: Control *n* = 9, Ipsi mEC *n* = 6.

Abbreviations: SLM, stratum lacunosum‐moleculare; SP, stratum pyramidale; SR, stratum radiatum.

These two findings, taken together, present a direct challenge to theories proposing that the 20–40 Hz band represents a distinct “slow gamma” rhythm driven by CA3 (Colgin et al. 2009; Belluscio et al. 2012) or lateral EC (Fernández‐Ruiz et al. 2021). The fact that power in this band is powerfully modulated by MEC input but is unaffected by the removal of its putative CA3 driver is fundamentally inconsistent with the multiplexing model. Instead, the widespread reduction across all layers following MEC inactivation supports the energy cascade framework, where disruption of the primary theta driver proportionally affects its harmonics throughout the circuit.

### 3.4 The 60–100 Hz Gamma Range

Gamma power (60–100 Hz) was most dramatically affected by MEC inactivation (Figure 3i), showing highly significant reductions in all layers: stratum lacunosum moleculare (71.1% reduction, *p* = 0.0001, p_FDR = 0.001, *d* = −2.95), stratum radiatum (46.1% reduction, *p* = 0.0007, p_FDR = 0.004, *d* = −2.28), and stratum pyramidale (53.0,0.001,% reduction, *p* = 0.001, p_FDR = 0.004, *d* = −2.23). In contrast, an LMM analysis of the CA3 inactivation alone revealed no significant change in gamma power in any layer (SP: −24.4% change, *p* = 0.228, Cohen's *d* = −0.99; SR: −20.9% change, *p* = 0.218, *d* = −1.02; SLM: −11.2% change, *p* = 0.521, *d* = −0.44; *n* = 3). The combined MEC + CA3 manipulation showed even more profound effects, with gamma power reductions of 76.9% in SLM (*p* = 0.0005, uncorrected; *d* = −4.55), representing the largest effect size observed in our study. These results align with the Interneuron Network Gamma (ING) model (Wang and Buzsáki 1996; Whittington et al. 2000), where gamma oscillations emerge from local interneurons driven by tonic excitation, with MEC providing critical theta‐paced excitatory drive (Mizuseki et al. 2009; Zhou et al. 2022).

### 3.5 Cross‐Frequency Power Changes

To directly test the hypothesis that the observed power reductions were interdependent, as predicted by the energy cascade framework, we next performed a correlation analysis on the magnitudes of power changes during inactivation. This analysis asks a critical question: did the amount of theta power that was lost predict the amount of gamma power that was lost?

For MEC inactivation, the results revealed a strong, positive relationship. The magnitude of harmonic power changes was significantly correlated with gamma power changes (*r* = 0.731, *p* = 0.0006), with harmonic changes explaining 53.4% of the variance in gamma power changes. Similarly, theta power changes showed a moderate positive association with gamma changes (*r* = 0.456, *p* = 0.057), accounting for 20.8% of the variance.

These findings strongly support a model where MEC inputs drive theta oscillations that subsequently organize higher frequency activity, consistent with the energy cascade framework and the ING model (Traub et al. 1996). In contrast, since CA3 inactivation produced no significant change in power in any of these frequency bands, a similar correlation analysis was not applicable. This lack of a primary effect from CA3 silencing further reinforces its minimal role in driving the broader spectral organization under these conditions. It indirectly affirms the lack of changes in place cell characteristics following Schaffer collateral knife cut lesions (Brun et al. 2002).

### 3.6 Cross‐Correlation Analysis of Power Spectral Density Across Layers

To investigate how energy flows through the hippocampal circuit, we examined the power correlations between the stratum pyramidale (SP) and stratum lacunosum‐moleculare (SLM) layers. This comparison is critical because SLM receives direct input from MEC via the perforant path, while SP contains the pyramidal cell bodies whose output represents the final product of hippocampal processing.

The multiplexing model predicts that CA3 drives CA1 ‘low gamma’ in SP, while MEC drives ‘high gamma’ in SLM, leading to selective disruption of these frequency‐ and layer‐specific channels. Based on this, the multiplexing model would predict selective disruption of correlations predominantly within these purportedly distinct high‐frequency bands (e.g., 20–40 Hz in SP, and 60–100 Hz in SLM). Conversely, the energy cascade model predicts that MEC inactivation should disrupt power correlations more broadly and proportionally across all frequency bands between these layers, reflecting an interdependent system rather than discrete, independently driven channels.

**FIGURE 4:**
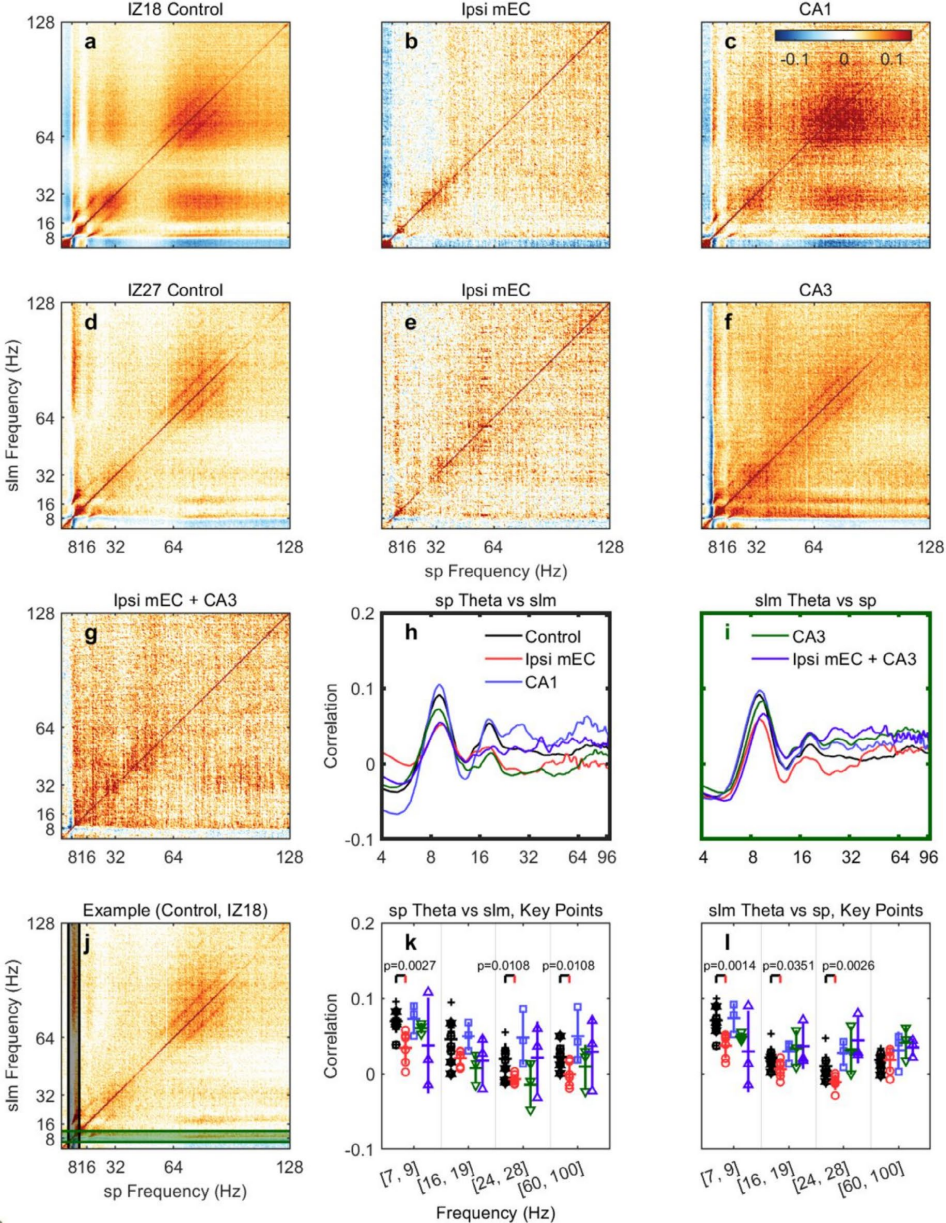
The cross‐correlation of PSD between sp. (x) and slm (y). (a) In the control case, there are correlations among the theta and its harmonics and the theta and gamma. The 1st and 2nd harmonics also correlate as shown by the off‐diagonal components at around 30 Hz. The correlations between the gamma and theta are indicated by the off‐diagonal components starting from around 60 Hz to 100 Hz, showing the theta is modulated by the gamma. Some weak correlations exist between the theta harmonics and gamma. (b) During the ipsi mEC inactivation, the correlations between the theta and gamma are gone. There exist very weak correlations between the theta and its 1st harmonics. This supports that the ipsi mEC inactivation cuts off the gamma rhythm and greatly suppress the theta activity in the brain. (c) When CA1 is inactivated, the correlation map is similar to that of the control case. This example is from IZ18. (d, e) Similar to (a, b), the off‐diagonal entries indicate that correlation between the theta and harmonic theta, theta and gamma are present in the control but not in the ipsi mEC inactivation. (f) Similar to (d), the CA3 inactivation does not cut the correlation between theta and harmonics theta, and theta and gamma. Also the slm theta correlates frequency components of sr more compared with sr theta to slm. (g) Specific correlation is not present during the ipsi mEC + CA3 inactivation. The animal is IZ27. (h–i) the average correlation for sp. theta versus slm and slm theta versus sp. (j) The corresponding regions selected to plot the correlation shown in (h) (black) and (i) (green). (k) The statistics of the cross correlation between the sp. theta (7–10 Hz) and slm key frequency intervals for the control, ipsi mEC, CA1, CA3, and ipsi mEC + CA3 inactivation. (l) The statistics of the cross correlation between the slm theta (7–10 Hz) and sp. key frequency intervals for all conditions. Please see Figure S4 for a zoomed‐in statistics figure.

Power‐power correlations were calculated as described previously (Masimore et al. 2004). In the control condition, the cross‐correlation matrices revealed a structured pattern of coupling between theta (~8 Hz), its harmonics (16 Hz, 24 Hz), and the 60–100 Hz gamma band (Figure 4a–c). Notably, our 1‐s analysis window was sufficient to resolve the expected power‐power coupling between theta and high‐frequency gamma, yet it revealed no evidence of significant coupling in the canonical “slow gamma” range (20–40 Hz). These overall patterns are consistent with similar analyses (Buzsáki et al. 2003; Masimore et al. 2004; Tort et al. 2010).

Statistical analysis employed linear mixed‐effects models with condition and frequency band as fixed effects and animal as a random effect. Based on our a priori hypothesis that MEC input coordinates CA1 oscillations, we designated Control vs. Ipsi MEC comparisons as planned comparisons (8 tests: 4 frequency bands × 2 directions) with false discovery rate (FDR) correction using the Benjamini‐Hochberg method.

MEC inactivation profoundly disrupted cross‐frequency power correlations within CA1. The organized correlational structure observed in control conditions was visibly lost following inactivation, and a subsequent quantification of specific frequency bands confirmed this widespread disruption of cross‐frequency coordination.

For SP theta → SLM correlations, MEC inactivation significantly reduced correlations at all frequencies tested. The theta–theta correlation decreased by 50.9% (Control: 0.070 ± 0.005 vs. Ipsi mEC: 0.034 ± 0.009; p_FDR = 0.007, *d* = −1.87). Notably, correlations with gamma frequencies not only decreased but reversed sign. In control conditions, when SP theta power increased, SLM gamma power also tended to increase (positive correlation). After MEC inactivation, this relationship reversed—increases in SP theta were associated with decreases in SLM gamma (negative correlation). The third harmonic region (24–28 Hz) showed a 135.8% reduction (Control: 0.020 ± 0.007 vs. Ipsi mEC: −0.007 ± 0.003; p_FDR = 0.017, *d* = −1.71) and high gamma (60–100 Hz) showed a 103.7% reduction (Control: 0.022 ± 0.005 vs. Ipsi mEC: −0.001 ± 0.007; p_FDR = 0.017, *d* = −1.54).

The reverse direction (SLM theta → SP correlations) showed similar disruption. The theta–theta correlation decreased by 44.9% (Control: 0.070 ± 0.005 vs. Ipsi mEC: 0.039 ± 0.006; p_FDR = 0.007, *d* = −2.15). 2nd harmonic (16–19 Hz) correlations decreased by 72.0% (p_FDR = 0.047, *d* = −1.30), and third harmonic correlations again reversed sign with a 242.5% reduction (Control: 0.008 ± 0.004 vs. Ipsi mEC: −0.011 ± 0.002; p_FDR = 0.007, *d* = −2.11). Only the SLM theta → SP high gamma correlation remained unchanged (p_FDR = 0.814).

Additional analyses examining input‐specific contributions with small sample sizes (*n* = 3) can only detect substantial effects and should be interpreted cautiously. As requested in review, CA3 inactivation alone showed theta–theta correlations (SP → SLM: 0.060 ± 0.004) that did not differ significantly from control (*p* = 0.354), while combined MEC + CA3 inactivation showed intermediate values (0.037 ± 0.037, *p* = 0.156 vs. control). With the current sample sizes, we cannot definitively assess whether CA3 manipulations alter cross‐frequency coordination.

The patterns in the correlation matrices present a key organizational principle that is inconsistent with the multiplexing/routing model. The apparent “gap” in correlations between ~34 and 64 Hz under control conditions reveals an absence in what is often described as “slow gamma” (while the same analyses were capable of replicating power correlations between theta and fast gamma). Caution should be taken in interpreting the widening of the higher harmonics as the fundamental theta frequency jitters; the bandwidth of its successive harmonics naturally widens, creating transition zones between them (i.e., if theta is 7–9 Hz, 2 Hz wide, the 3rd harmonic will be 28–36 Hz, or 8 Hz wide).

### 3.7 Effects of Inactivation on Coherence and Theta Phase

Inter‐laminar coherence provides a critical test for distinguishing between multiplexing and energy cascade frameworks. Multiplexing theories predict that different frequency bands should show anatomically segregated coherence patterns and that circuit perturbations should selectively disrupt these specific channels. In contrast, the energy cascade model predicts that perturbations should broadly disrupt inter‐laminar coordination across frequencies, reflecting a deeply integrated system. To test these competing predictions, we analyzed coherence between the stratum lacunosum‐moleculare (SLM) and other CA1 laminae, designating Control vs. Ipsi mEC comparisons as our planned comparisons with FDR correction.

**FIGURE 5:**
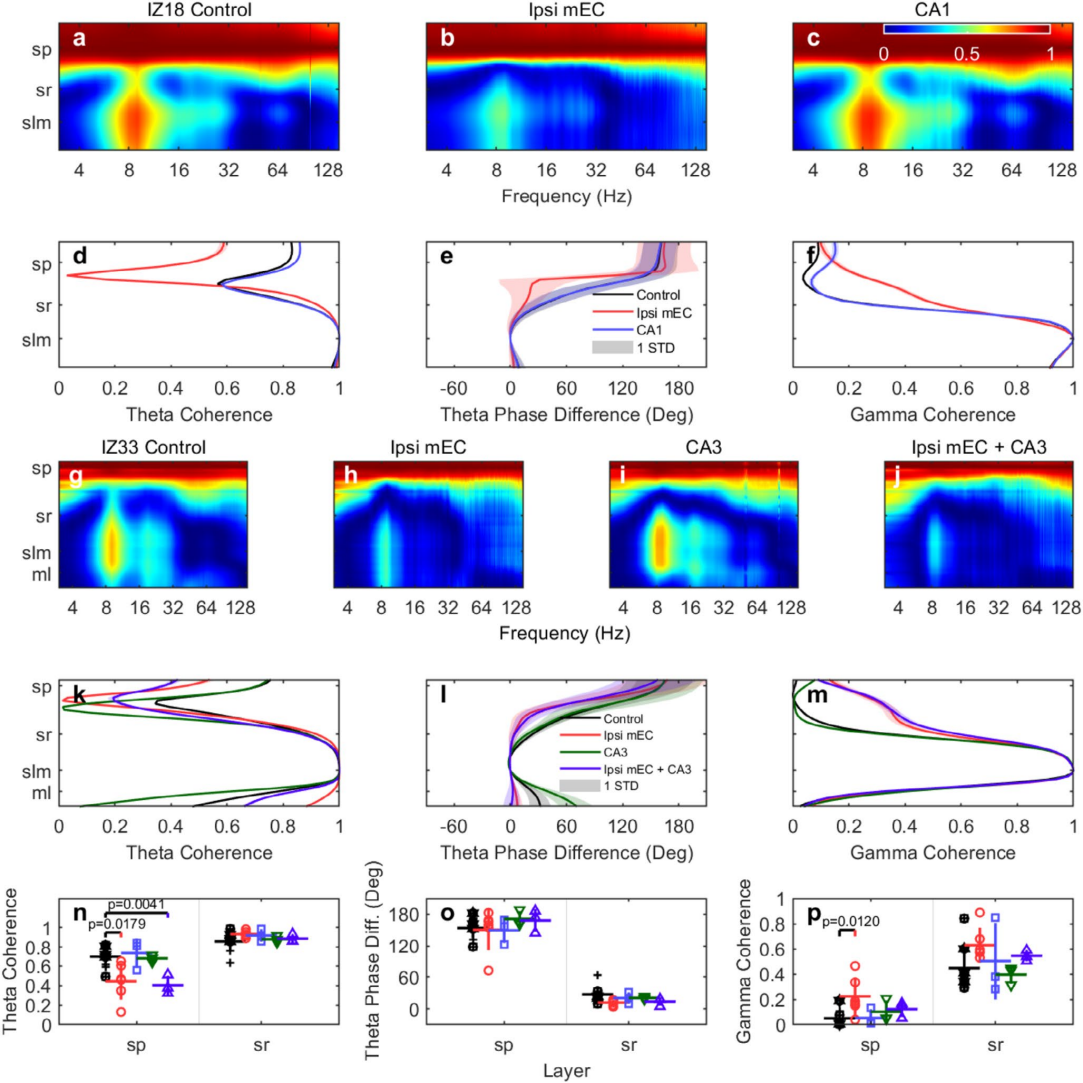
Inter‐laminar coherence analysis across CA1 layers during different inactivation conditions. Coherence heatmaps (panels a–c, g–j): Color intensity represents coherence magnitude across frequencies (4–128 Hz, *x*‐axis) and CA1 layers (*y*‐axis). Line plots (panels d–f, k–m): Coherence or phase values plotted directly on the *x*‐axis for specific frequency bands, with different conditions shown as colored lines. Shaded error bars for the phase difference are 1 standard deviation. Shaded regions for coherence indicate the 95% confidence interval calculated via the Fisher *z*‐transform. Note: Due to the high statistical power provided by the large number of averaged segments (*L* > 500), these confidence intervals are very narrow and may be visually indistinguishable from the coherence trace itself. Upper panels (IZ18): (a–c) Coherence heatmaps with SP as reference layer. (a) Control shows theta coupling, harmonics, and gamma coherence. (b) MEC inactivation weakens theta/harmonic coupling and eliminates gamma coherence, creating a bottleneck (bottleneck refers to a frequency range where energy transfer between scales is temporarily constrained, a common feature in energy cascade systems) between SP–SR. (c) CA1 inactivation shows minimal changes. (d) Theta coherence quantification (SLM reference) shows MEC‐specific reduction in SLM‐SP coupling. (e) Theta phase differences reveal disrupted phase relationships during MEC inactivation. (f) Gamma coherence (SLM reference) paradoxically increases between SP–SR during MEC inactivation. Middle panels (IZ33): (g–j) Coherence heatmaps with SP as reference layer across four conditions. (g) Control preserves harmonic and gamma patterns. (h) MEC inactivation disrupts these patterns. (i) CA3 inactivation preserves most coherence features. (j) Combined MEC + CA3 inactivation shows strongest disruption. (k–m) Quantitative comparisons across all conditions for theta coherence, phase differences, and gamma coherence respectively. Statistical summaries (n–p): Group‐level statistics for theta coherence, phase differences, and gamma coherence across layers and conditions. Significance levels are indicated by *p*‐values. (d, f, k, m): For each experimental condition, 95% confidence intervals for the coherence estimates were calculated separately via the Fisher *z*‐transform (Bendat and Piersol 2011). The number of segments (L) used in this calculation was determined by the total degrees of freedom available for each respective condition. Please see Figure S5 for a zoomed‐in statistics figure.

A qualitative overview of the coherence‐by‐depth plots revealed a clear disruption of the circuit's organization following MEC inactivation. In control conditions, a clear laminar pattern was visible, with weak theta coherence between SP and SR, which progressively increased toward SLM, where strong gamma coherence also emerged (Figure 5a,g). During MEC inactivation, this entire laminar pattern was disrupted: both the region of weak SP‐SR coherence and the strong gamma coherence in SLM became less distinct, and the theta harmonic structure was less evident (Figure 5b,h).

Quantitative analysis revealed that MEC input is essential for maintaining long‐range theta‐frequency coordination. In the theta band (7–10 Hz), MEC inactivation significantly reduced SLM‐SP coherence by 35.8% (Control: 0.682 ± 0.039 vs. Ipsi mEC: 0.438 ± 0.094; *t*(11) = 2.779, *p* = 0.018, p_FDR = 0.036, Cohen's *d* = −1.46), but not SLM‐SR coherence (Control: 0.856 ± 0.040 vs. Ipsi mEC: 0.935 ± 0.016; 9.2% increase; *t*(11) = −1.500, *p* = 0.162, p_FDR = 0.162, Cohen's *d* = 0.95) as shown in Figure 5n.

In contrast, MEC inactivation led to a massive and significant increase in local gamma coherence (60–100 Hz). SLM‐SP gamma coherence increased from near‐chance levels to substantial coupling, a 417.8% increase (*t*(11) = −3.004, *p* = 0.012, p_FDR = 0.036, Cohen's *d* = 1.54, very large effect). The coherence between SLM and SR did not differ significantly between the Control (0.465 ± 0.061) and Ipsi mEC (0.651 ± 0.064) conditions. While a numerical increase of 40.1% was observed, corresponding to a large effect size, this difference was not statistically significant after correction for multiple comparisons (*t*(11) = −2.001, *p* = 0.071, p_FDR = 0.094, Cohen's *d* = 1.17), as shown in Figure 5p.

Additional analyses examining the CA3 inactivation (*n* = 3) were performed using Linear Mixed‐Effects Models. These analyses revealed no statistically significant effects on inter‐laminar coherence or phase difference (although these results should be interpreted with caution due to the low sample size). Specifically, theta coherence between layers was not significantly changed (SLM‐SP: *p* = 0.589, *d* = −0.37; SLM‐SR: *p* = 0.686, *d* = 0.27), nor was gamma coherence (SLM‐SP: *p* = 0.531, *d* = 0.43; SLM‐SR: *p* = 0.301, *d* = −0.80). Finally, theta phase differences also remained unchanged (SLM‐SP: *p* = 0.840, *d* = 0.13; SLM‐SR: *p* = 0.668, *d* = 0.29).

While MEC inactivation significantly disrupted the consistency of the theta phase relationship (i.e., coherence), it did not systematically alter the average phase difference between CA1 laminae (Figure 5e,l,o). Although the SLM‐SR phase relationship showed a substantial, non‐significant trend toward reduction (Control: 26.5° ± 5.9° vs. Ipsi mEC: 11.2° ± 2.5°; 15.3° or 57.8% reduction; *t*(11) = 1.969, *p* = 0.075, p_FDR = 0.149, Cohen's *d* = −1.24), the primary SLM‐SP phase relationship remained remarkably stable (Control: 153.2° ± 7.2° vs. Ipsi mEC: 147.4° ± 19.4°; 5.8° reduction; *t*(11) = 0.333, *p* = 0.745, p_FDR = 0.745, Cohen's *d* = −0.17). This dissociation suggests that while MEC input is critical for maintaining the cycle‐by‐cycle consistency of theta synchronization, the fundamental phase alignment across the CA1 circuit is resilient to disruption.

### 3.8 Effects of Inactivation on Cross‐Frequency Coupling

Bicoherence analysis across different layers and conditions provides insights into nonlinear interactions between different frequency components of hippocampal activity (Bullock et al. 1997; Aru et al. 2014; Zhou et al. 2019; Sheremet et al. 2020). Cross‐frequency phase coupling was assessed using bicoherence analysis following Hagihira et al. (2001), which quantifies phase coupling between three frequency components (f_1_, f_2_, f_1_ + f_2_) and ranges from 0 (no coupling) to 1 (perfect phase coupling). We computed theta–theta coupling (9 Hz with its harmonics at 18 Hz and 27 Hz) and theta–gamma coupling (9 Hz, gamma frequencies, 9 + gamma Hz) for each CA1 layer.

Statistical analysis employed linear mixed‐effects models with condition and layer as fixed effects and animal as a random effect. Based on our a priori hypothesis that MEC input coordinates CA1 nonlinear coupling, and to test whether MEC and CA3 inputs provide redundant versus additive contributions to phase coupling, we designated Control versus [Ipsi mEC, mEC + CA3] comparisons as planned comparisons (12 tests: 2 coupling types × 3 layers × 2 conditions) with FDR correction using the Benjamini‐Hochberg method.

**FIGURE 6:**
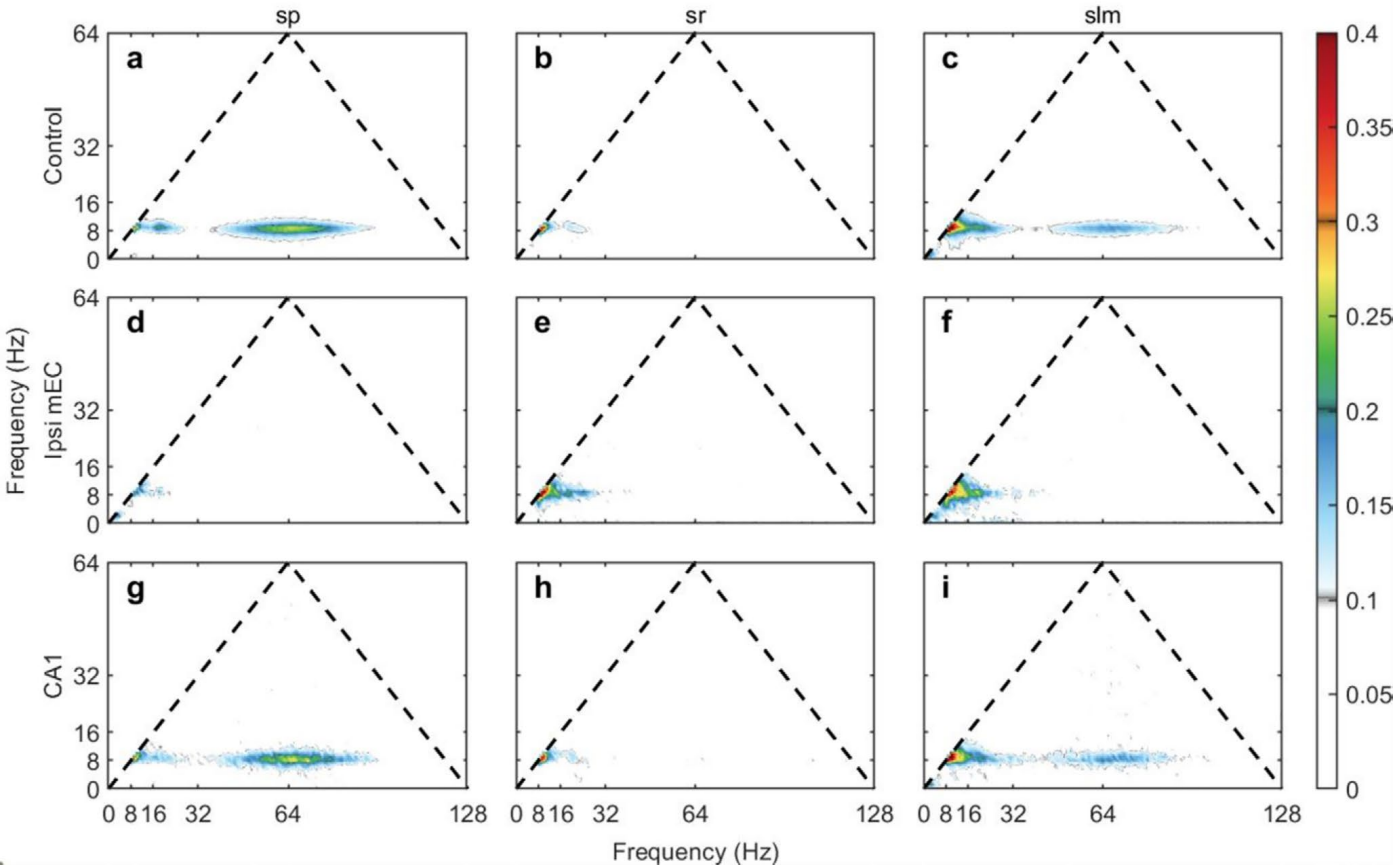
The bicoherence of different layers during the control, ipsi mEC, and CA1 inactivation. (a) The bicoherence of control, sp. shows coupling of 9, 18, 27 Hz (theta–theta) as well as gamma (theta‐gamma) with these frequency components. (b) For control, sr, the coupling of theta and its harmonics exists, but is lower compared with a. The theta‐gamma coupling is much weaker. (c) For control, slm, the theta–theta coupling is stronger compared with a, but the theta‐gamma coupling is weaker. (d, f) During the ipsi mEC inactivation, the theta–theta coupling is weaker than (a) and (c). The theta‐gamma coupling is not seen in the current color bar. (e) Still, the theta‐gamma coupling is not present, but the theta–theta coupling tends to become stronger. (g–i) During the CA1 inactivation, the patterns of bicoherence are not significantly different than the corresponding control maps. This animal is IZ18. Please see Figure S1 for a zoomed‐in depiction of the lower frequency range encompassing the harmonics.

**FIGURE 7:**
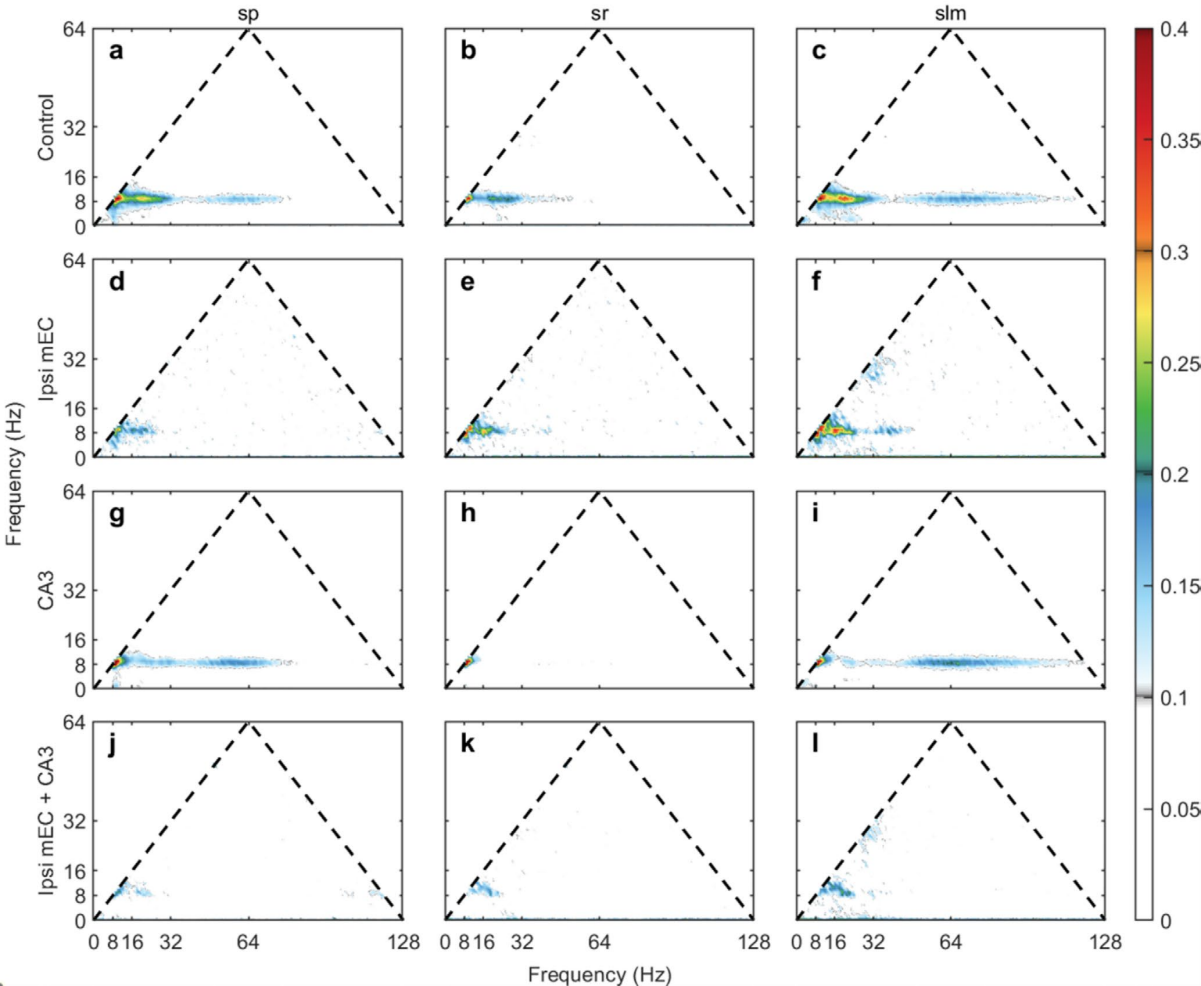
The bicoherence of different layers during the control, ipsi mEC, CA3, and ipsi mEC + CA3 inactivation. The control condition (a–c) and ipsi mEC inactivation (d–f) show similar relationships as (Figure 6a–f). (g–i) During the CA3 inactivation, similar to the control condition, the theta‐gamma bicoherence exists, while the theta–theta is weaker. (j–l) During the ipsi mEC + CA3 inactivation, similar to the ipsi mEC inactivation, the theta‐gamma bicoherence is not seen. The theta–theta bicoherence is even weaker. This animal is IZ33. Please see Figure S2 for a zoomed‐in depiction of the lower frequency range encompassing the harmonics.

**FIGURE 8:**
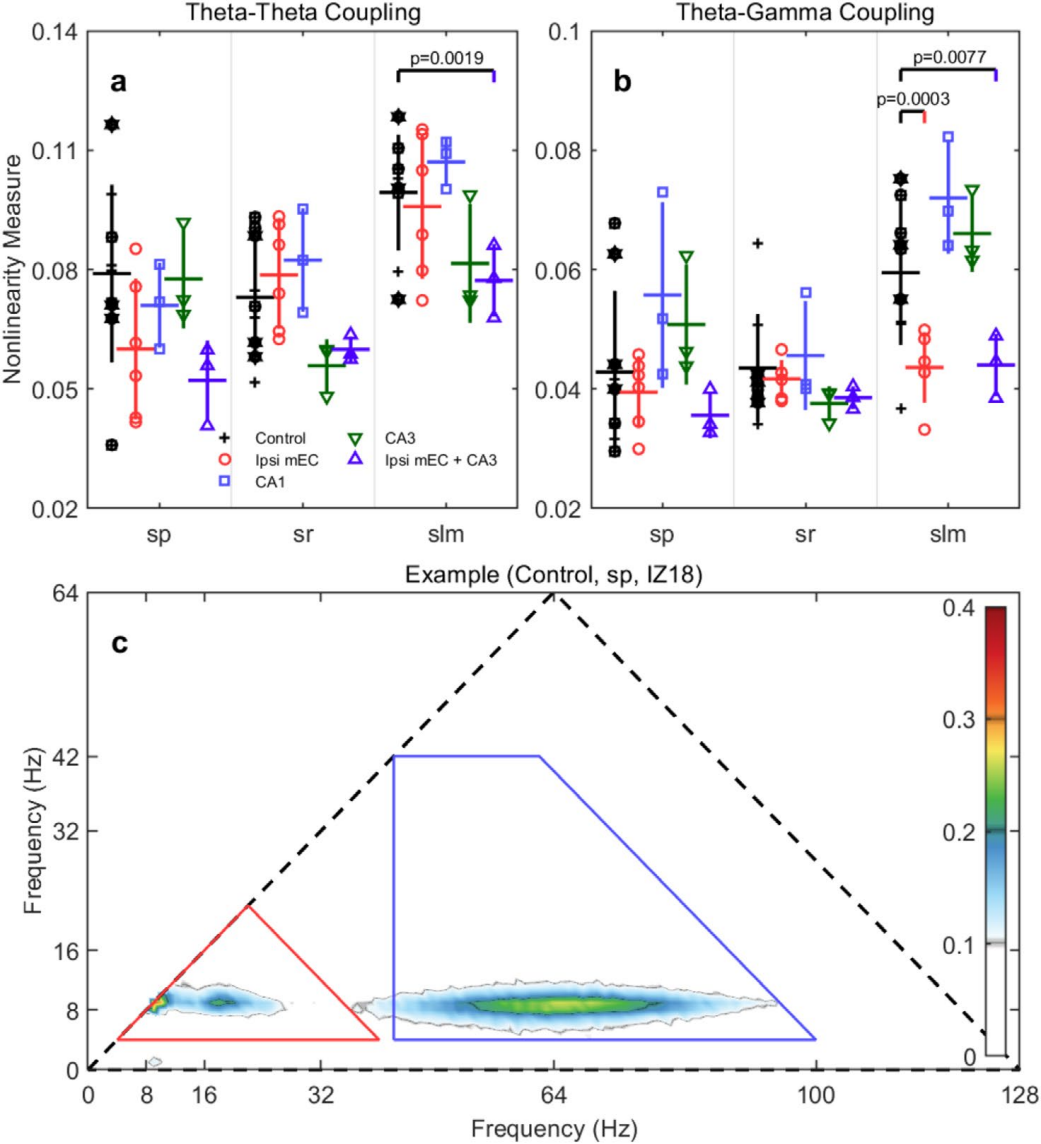
The bicoherence statistics of the theta–theta and theta‐gamma coupling. (a) The overall theta–theta coupling by summing up and normalizing the values in the red triangle in (c). (b) The overall theta‐gamma coupling by summing up and normalizing the values in the blue trapezoid in (c). (c) An example showing the regions used to estimate the theta–theta coupling and theta‐gamma coupling. The red triangle is bounded by frequency interval [4, 40] Hz. The blue trapezoid is bounded by frequency interval [42, 100] Hz.

MEC manipulations produced selective, layer‐specific disruptions in cross‐frequency phase coupling within CA1. Of 12 planned comparisons, 3 survived FDR correction (25% survival rate), all localized to the stratum lacunosum‐moleculare where entorhinal fibers terminate (Figures 6, 7, 8).

Theta–theta coupling (harmonic interactions) showed selective vulnerability to combined manipulations. MEC inactivation alone produced no significant changes in theta–theta coupling across layers (SLM: −1.8%, *p* = 0.355, p_FDR = 0.707). However, combined MEC + CA3 inactivation significantly reduced theta–theta coupling in SLM by 22.1% (Control: 0.0993 ± 0.0028 vs. mEC + CA3: 0.0773 ± 0.0027; *t* = 4.573, *p* = 0.0019, p_FDR = 0.0115, Cohen's *d* = −3.20). This very large effect size represents the strongest effect observed across all analyses in this study, suggesting that disrupting both entorhinal and CA3 inputs is required to substantially affect harmonic phase relationships in the distal dendritic layer.

Theta‐gamma coupling showed robust disruption from MEC manipulation. MEC inactivation alone significantly reduced theta‐gamma coupling in SLM by 26.7% (Control: 0.0595 ± 0.0026 vs. Ipsi mEC: 0.0437 ± 0.0009; *t* = 3.981, *p* = 0.0003, p_FDR = 0.0038, *d* = −2.79). Combined MEC + CA3 inactivation produced a nearly identical effect in SLM (−26.1%, *p* = 0.0077, p_FDR = 0.0310, *d* = −2.66), indicating that CA3 inputs do not significantly contribute to theta‐gamma phase coupling in this layer and that MEC provides the primary drive for cross‐frequency phase coordination.

Other planned comparisons did not reach statistical significance after FDR correction. Theta‐gamma coupling in SP and SR layers showed minimal effects from either manipulation, while theta–theta coupling in SP and SR layers remained largely intact. As previously reported (Sheremet et al. 2019), there was a significant laminar gradient in bicoherence values, with the highest values in the lacunosum moleculare and the lowest in the pyramidal cell layer. As suggested by the reviewer, we conducted additional exploratory analyses on smaller cohorts involving the inactivation of CA1 or CA3 alone (*n* = 3 for each). While the statistical power of these experiments is limited, they reveal a functional dissociation.

First, CA1 inactivation resulted in statistically significant increases in theta‐gamma coupling in both stratum pyramidale (SP; +30.0%, *p* = 0.026, p_FDR = 0.033) and stratum lacunosum‐moleculare (SLM; +20.9%, *p* = 0.028, pFDR = 0.05). This suggests that removing the influence of CA1 pyramidal cells unmasks or enhances local gamma‐generating microcircuits. In contrast, the inactivation of CA3 alone produced a more isolated effect, with only theta–theta coupling in the SLM showing a significant reduction (−17.9%, *p* = 0.012, pFDR = 0.016).

These biocherence results reveal a clear double dissociation in the mechanisms coordinating cross‐frequency coupling in CA1. Theta‐gamma phase coupling in the LM appears to be driven primarily by entorhinal inputs; it is robustly disrupted by MEC inactivation alone and is not further affected by the additional silencing of CA3. Conversely, theta–theta harmonic coupling emerges as a far more resilient network property. This feature is only significantly reduced following the combined disruption of both MEC and CA3 inputs, suggesting the fundamental, non‐linear waveform of the theta rhythm is a highly robust feature stabilized by convergent inputs from multiple pathways.

## 4 Discussion

Here we tested the competing predictions of the gamma multiplexing/routing and energy cascade models by analyzing the effects of circuit perturbations on hippocampal oscillations. Our findings consistently favor an energy cascade framework. Specifically, we found that: (1) MEC inactivation caused broadband, proportional power reductions across the spectrum, not the selective disruption of a “fast gamma” channel predicted by multiplexing/routing; (2) the 20–40 Hz band was not selectively eliminated by its putative CA3 driver and was confirmed by bicoherence to be a theta harmonic, contrary to the “slow gamma” hypothesis; and (3) cross‐frequency and cross‐layer coupling patterns revealed a deeply integrated system, not a set of segregated channels. Below, we discuss how these findings systematically contradict the tenets of multiplexing and support a unified, interdependent view of hippocampal oscillatory organization.

### 4.1 MEC Integrity and Hippocampal Theta

While Zutshi et al. (2022) first demonstrated that MEC inactivation reduces oscillatory power in CA1, our reanalysis through the lens of energy cascade theory reveals new insights into its organizational principles. Consistent with Zutshi et al. (2022), our analyses affirm that MEC inactivation significantly reduces hippocampal theta power, especially in the stratum lacunosum‐moleculare. However, theta persisted at reduced levels, indicating that while MEC input is critical, it is not the sole source of the rhythm (Petsche et al. 1962; Mitchell and Ranck 1980; Alonso and García‐Austt 1987a, 1987b; Kocsis et al. 1999; Buzsaki 2002). This result, a significant but incomplete reduction of the system's primary low‐frequency driver, is the first piece of evidence for a resilient, yet highly interdependent system. It establishes the initial impact of the perturbation which, according to the energy cascade model, should then propagate proportionally to higher frequencies.

Moreover, the layer‐specific reduction, being most pronounced in slm, highlights how MEC input powerfully influences the distal dendrites of CA1 pyramidal cells. This aligns with anatomical data on entorhinal projections (Amaral and Witter 1989; Witter et al. 2000) and supports the notion that MEC input modulates dendritic integration in CA1 (Mizuseki et al. 2009), even though CA1 remains partially autonomous (Zutshi et al. 2022; Sloin et al. 2024).

### 4.2 Synaptic Input and Gamma Oscillations

Consistent with Zutshi et al. (2022), we observed a marked reduction in gamma amplitude during medial entorhinal cortex (MEC) inactivation, but not when CA1 principal cells were silenced. This result strongly supports an Interneuron Network Gamma (ING) model, where local interneuron networks generate gamma oscillations in response to adequate excitatory drive (Whittington et al. 1995; Wang and Buzsáki 1996). Our findings identify MEC as providing a substantial portion of this excitation, but this does not imply a “gamma relay” hypothesis whereby oscillations are inherited wholesale from upstream regions (e.g., Colgin et al. 2009; Fernández‐Ruiz et al. 2021).

Instead, our findings favor a model in which MEC input modulates the excitability of local hippocampal circuits that then generate gamma intrinsically (Csicsvari et al. 2003; Pernía‐Andrade and Jonas 2014). This interpretation is well supported by the known microcircuitry: interneurons in the stratum lacunosum‐moleculare can receive direct perforant path inputs (Kajiwara et al. 2008), and other pathways like the alvear pathway activate multiple CA1 interneuron subtypes in a layer‐specific manner (Deller et al. 1996; Bell et al. 2021). The partial preservation of gamma after MEC inactivation further suggests contributions from these and other excitatory pathways (Amaral and Witter 1989; Wouterlood et al. 1990; Vertes 1992).

Ultimately, this core finding, that local gamma power scales with the available excitatory drive from multiple potential sources, corroborates a local ING mechanism over a simple relay model and serves as crucial evidence for the energy cascade framework, where the energy of the broader network state dictates the power of local, high‐frequency activity.

It is important to note that the energy cascade framework applies broadly to high‐frequency activity in the gamma range, encompassing both sustained oscillatory periods and more transient broadband phenomena. This inclusive approach recognizes that hippocampal ‘gamma’ likely represents a spectrum of activity types, from rhythmic interneuron synchronization to brief bursts of desynchronized spiking, all of which can contribute to spectral power in this frequency range. Rather than requiring strict periodic criteria to qualify as ‘oscillations,’ a cascade model accommodates the complex, dynamic nature of gamma activity as it emerges from continuous energy transfer processes across frequency scales.

### 4.3 Comparing Multiplexing/Routing and Energy Cascade Models

The multiplexing/routing model proposes that distinct gamma sub‐bands function as discrete, multiplexed channels routing information between different circuits in the entorhinal cortex and hippocampus network (Colgin et al. 2009; Fernández‐Ruiz et al. 2017, 2021). Our findings challenge this view on several fundamental grounds. First, our several quantitative analyses could not detect discrete gamma sub‐bands. Consistent with recent findings that challenge rigid band definitions (Douchamps et al. 2024), our bicoherence analyses revealed broad, multi‐frequency interactions, characterizing a unified process rather than segregated channels.

Second, our MEC inactivation results undermine the notion of the entorhinal cortex as a singular “broadcaster” of specific gamma frequencies. While MEC input is critical, the fact that theta and gamma rhythms persisted (albeit at reduced power) demonstrates the resilience and distributed nature of rhythm generation within the hippocampus.

Third, our findings from the CA3 inactivation provide a direct refutation of the hypothesis that CA3 is the primary generator of a distinct “slow gamma” oscillation. The multiplexing model predicts that silencing CA3 should selectively eliminate that slow gamma band. In contrast, we found that CA3 inactivation alone produced no significant reduction in power, a result that is fundamentally inconsistent with it being a driver of gamma. The marginal influence of CA3 on CA1 place cells has been noted earlier, in which CA1 neurons were able to develop sharp and stable place fields, sans CA3 input (Brun et al. 2002). However, this does not imply the CA3 input is irrelevant. Our bicoherence analysis revealed a more nuanced and critical role: while theta‐gamma coupling in the LM was dependent on MEC alone, the robust theta–theta harmonic structure was only disrupted when both MEC and CA3 inputs were silenced simultaneously. This suggests that the non‐sinusoidal shape of the theta rhythm, the ultimate source of the harmonics, is a highly resilient network property, stabilized by convergent and potentially redundant inputs from both major pathways. Tenably, this would operate through the interaction of two spatially segregated theta resonances in the dendrite and soma of CA1 neurons (Kamondi et al. 1998; Hu et al. 2009; Losonczy et al. 2010; Noguchi et al. 2023; Liao et al. 2024). Rather than generating a separate “slow gamma” channel, CA3 input appears to influence MEC activity to alter theta waveform shape and the fundamental theta rhythm.

Fourth, the dendrites of CA1 neurons act as a low‐pass filter for synaptic inputs. Consequently, while theta‐modulated gamma arrives at the dendrites, the fast oscillatory components themselves are filtered out as they propagate to the cell body: “*While this finding supports the efficiency of gamma‐frequency inputs in distal signaling, it presents a conundrum for the notion that high‐frequency inputs from the CA3 region or the direct entorhinal input can entrain high‐frequency CA1 output*” (Vaidya and Johnston 2013, 8). In essence, the same dendritic properties that make the neuron an effective integrator of theta‐paced activity also make it an ineffective conduit for high‐frequency gamma signals. This presents a fundamental challenge to any model that relies on the direct, feed‐forward transmission of gamma oscillations from input to output in CA1.

Fifth, the premise of gamma‐as‐router is conceptually flawed. Because gamma power is itself phase‐locked to the slower theta rhythm, any putative “routing” would ultimately be governed by theta's timescale. This challenges the idea that gamma independently routes information. Notably, many studies supporting gamma routing omit a detailed analysis of theta or theta harmonics, overlooking the primary organizing influence on gamma's structure.

Finally, this framework is challenged by the results of principal cell inactivation. The foundational literature posits that gamma's primary role is to coordinate CA1 principal cell assemblies for information routing (Colgin et al. 2009; Fernández‐Ruiz et al. 2017). This creates a significant paradox: if gamma, imposed by external regions onto CA1, has the essential function of coordinating principal cell firing, why is coordinated network activity preserved when the inputs are silenced? This finding is reinforced by Zutshi et al.'s (2022) own conclusion that “the CA1 network can induce and maintain coordinated cell assemblies with minimal reliance on its inputs.” The preservation of network coordination in the absence of the very cells supposedly being coordinated suggests that gamma functions more as an emergent property of local interneuron dynamics, rather than as a discrete, top‐down coordination mechanism. While bilateral inactivation would be required for a definitive conclusion, this result is far more consistent with an energy cascade model where gamma arises from local circuit properties.

### 4.4 Cross‐Frequency Power Correlations Reveal a Unified and Integrated System

Our cross‐frequency power correlation analyses provide further, powerful evidence against the core organizational principles of the multiplexing/routing theory. The results strongly support the energy cascade model by demonstrating that MEC inactivation disrupts power correlations across the entire frequency spectrum, rather than selectively affecting a single gamma channel as predicted by multiplexing. Critically, our analyses of the 20–40 Hz band, which we describe as theta harmonics, showed the same uniform reduction in correlations as other frequencies, suggesting this band behaves as an integral component of the broader spectral cascade rather than a distinct, independently driven ‘slow gamma’ oscillation.

Furthermore, the specific patterns of correlation argue for a deeply integrated, not segregated system. We observed strong cross‐layer correlations between different frequency bands (e.g., low‐frequency components in SLM correlating with high‐frequency components in SP), which directly contradict the multiplexing prediction of layer‐specific, anatomically segregated channels. Instead, these cross‐layer relationships support an integrated system with energy transfer across both frequencies and spatial scales.

Most strikingly, the reversal of some gamma correlations (from positive to negative) with MEC inactivation indicates not merely a loss of coordination but an active reorganization of cross‐frequency relationships. This reflects the emergence of competing local dynamics when MEC's organizing influence is removed—a complex, non‐linear phenomenon that cannot be explained by a simple, independent channel model. In sum, the coordinated reduction in correlations across theta, its harmonics, and gamma demonstrates that these oscillatory components form an integrated system, where MEC input provides a critical energetic contribution that propagates throughout the circuit.

In contrast to the multiplexing/routing model, the energy cascade model (Buzsaki and Draguhn 2004; Sheremet, Qin, et al. 2018) posits that hippocampal oscillations arise not from discrete oscillators, but through an interdependent energy transfer from slower, larger‐scale rhythms (theta) to faster, smaller‐scale oscillations (gamma), akin to a weakly turbulent medium. This framework makes several predictions that the current data support. First, the theory is consistent with a resilient, self‐organizing system (Kelty‐Stephen et al. 2013); accordingly, we found that rhythms persisted (albeit attenuated) after MEC inactivation. Second, the theory predicts broad cross‐scale interactions; our bicoherence results (Figures 6a–c and 7a–c) provide direct evidence for this multi‐frequency coupling. Finally, the theory predicts that the strongest effects should be observed where the driving energy enters the system; indeed, the effects of inactivation on coupling were most pronounced in the stratum lacunosum‐moleculare, where entorhinal inputs terminate.

Notably, the observation of strong theta‐gamma bicoherence (Figures 6 and 7) indicates consistent phase relationships between theta and gamma frequencies, while our power‐power correlation analyses (Figure 4) demonstrate positive correlations between theta and gamma power. These patterns support the energy cascade framework: if gamma were independently generated discrete oscillations that are merely temporally interleaved as multiplexing theory suggests, we would expect both weak phase coupling AND uncorrelated power fluctuations with theta. Instead, the robust bicoherence combined with positive power correlations suggests gamma emerges from theta dynamics through systematic phase relationships and coordinated amplitude modulation. Overall, these observations favor an energy‐transfer framework over one predicated on multiple, function‐specific gamma sub‐bands.

### 4.5 Re‐Evaluating Regionally Localized Versus Globally Integrated Oscillations

A traditional view of hippocampal function posits that oscillations like theta and gamma originate in distinct subregions as independent local oscillators, a perspective that aligns with classical notions of specialized compartments. Our findings, however, directly challenge this localized view. We argue for a globally integrated perspective, in which hippocampal rhythms are not spatially segregated but operate as an interdependent, cross‐scale process. Just as modern neuroscience views cognitive functions as emerging from distributed networks rather than from single brain regions, our data indicate that hippocampal oscillations emerge from a similarly integrated network.

Key evidence for this integrated perspective comes from the power‐law scaling observed in hippocampal LFPs, which is indicative of scale‐free, turbulent‐like dynamics that are not confined to specific bands. This view is further supported by findings that theta‐gamma coupling strengthens with overall network activation, such as running speed, suggesting a globally coordinated process rather than the interaction of multiple, independent local oscillators (Sheremet et al. 2019). Consequently, the common practice of parsing gamma into narrow sub‐bands (e.g., Lopes‐Dos‐Santos et al. 2018; Zhang et al. 2019) has limited utility, particularly as recent, detailed analyses fail to confirm the existence of genuinely separate rhythms (Douchamps et al. 2024).

Recent work by Douchamps et al. (2024) provides complementary evidence against rigid, discrete gamma sub‐bands, approaching the question from a different analytical scale. While our study characterizes the overall turbulent flow of neural oscillations across frequency bands, Douchamps et al. (2024) focus on individual eddies within this flow. These transient gamma elements occur at diverse frequencies and phases. Their finding that these elements are broadly distributed across frequencies and phases, yet still carry behavioral information, aligns perfectly with turbulence physics principles that inform our cascade model. In turbulent systems, individual eddies appear discrete and chaotic at small scales while collectively forming a continuous energy cascade across frequencies. Similarly, the hippocampal oscillatory system exhibits both properties simultaneously: discrete events at fine timescales (as shown by Douchamps et al. 2024) and continuous, structured energy transfer across frequency scales (as demonstrated in our analyses). This multi‐scale perspective resolves the apparent contradiction between discrete events and continuous cascade, showing how these different analytical approaches reveal complementary aspects of the same underlying phenomenon—a complex, turbulent oscillatory system that transfers energy and information across multiple frequency scales.

### 4.6 Limitations and Considerations

Several important limitations regarding the interpretation of spectral analyses should be considered. The term ‘oscillation’ itself presents a fundamental challenge due to the discrepancy between the assumptions of spectral analysis and the complex nature of brain activity. This issue, historically termed the “Fourier Fallacy,” (Jasper 1948; yet equally applicable to wavelet analyses) highlights the flawed belief that a peak in a power spectrum corresponds to a literal, continuous sine wave being generated by a distinct biological oscillator. This gives the impression that the LFP is precisely divisible; however, as noted by Theodore Bullock, calling brain signals “oscillations” or “rhythms” is an imperfect analogy. They are not truly periodic like a clock, and it is fundamentally difficult to prove that any observed synchrony is statistically significant, as we cannot create a reliable null hypothesis for what “chance” synchrony looks like in the brain's complex and unknown physical structure (Bullock 1990).

This ambiguity is particularly problematic when considering specific confounding signals. For instance, the non‐sinusoidal shape of a genuine rhythm can generate harmonics that are easily misidentified as independent oscillations (Scheffer‐Teixeira and Tort 2016; Zhou et al. 2019). Similarly, spike bleed‐through from individual neurons can contribute power to the same spectral range as an underlying oscillatory process (Scheffer‐Teixeira et al. 2013; Pesaran et al. 2018). Therefore, no single frequency band can ever be exclusively described by a single neurobiological process.

These interpretational challenges are compounded by the practical choices made during analysis. It is worth noting that using smaller temporal windows increases frequency bleeding (e.g., spikes contributing to gamma or harmonics appearing as a dissociable “slow gamma”) due to the time‐frequency uncertainty principle (Gabor 1946; Tallon‐Baudry et al. 1997; Harris 2005). Ultimately, while the algorithmic decimation into sine waves is a necessary simplification for LFP quantification, it risks misclassifying these confounding signals, as it forces a discrete label onto what is fundamentally a continuous and analog biological process.

## 5 Conclusion

Our results establish two fundamental principles of hippocampal oscillatory organization: the dynamics are multi‐scale, and the underlying circuitry is highly interdependent and resilient. We demonstrate that while the medial entorhinal cortex input is an essential modulator, the hippocampus retains the intrinsic capacity to maintain both theta and gamma rhythms even when both MEC and CA3 inputs are silenced. These findings provide direct support for an energy‐transfer or “turbulence” framework over theories based on discrete gamma channels. By integrating evidence from circuit perturbations, laminar analyses, and cross‐frequency coupling, we demonstrate that hippocampal computation emerges from a fluid interplay of local and long‐range inputs, not from segregated, frequency‐bound channels. Future work extending this framework may further clarify how these multi‐scale dynamics contribute to spatial navigation, memory formation, and other cognitive functions.

## Funding

This work was supported by the National Institute of Mental Health, MH109548, MH126236. National Institute on Aging, AG055544. Evelyn F. McKnight Brain Research Foundation.

## Conflicts of Interest

The authors declare no conflicts of interest.

## Supporting information


**Data S1:** Supporting Information.

## Data Availability

The data that support the findings of this study are available in Buzsaki Lab Webshare at https://buzsakilab.nyumc.org/datasets/ZutshiI/Neuron2021. These data were derived from the following resources available in the public domain: Buzsaki Laboratory, https://buzsakilab.nyumc.org/datasets/ZutshiI/Neuron2021.
