# Supplementary material for: Entorhinal Silencing Reveals Energy Cascade Organization of Hippocampal Oscillations
Source: Hippocampus. 2025 Dec 8;36(1):e70050. doi: 10.1002/hipo.70050 (PMC12685368; doi:10.1002/hipo.70050)
Supplement: Supplementary file 1 — Data S1: Supporting Information. [file HIPO-36-0-s001.docx]

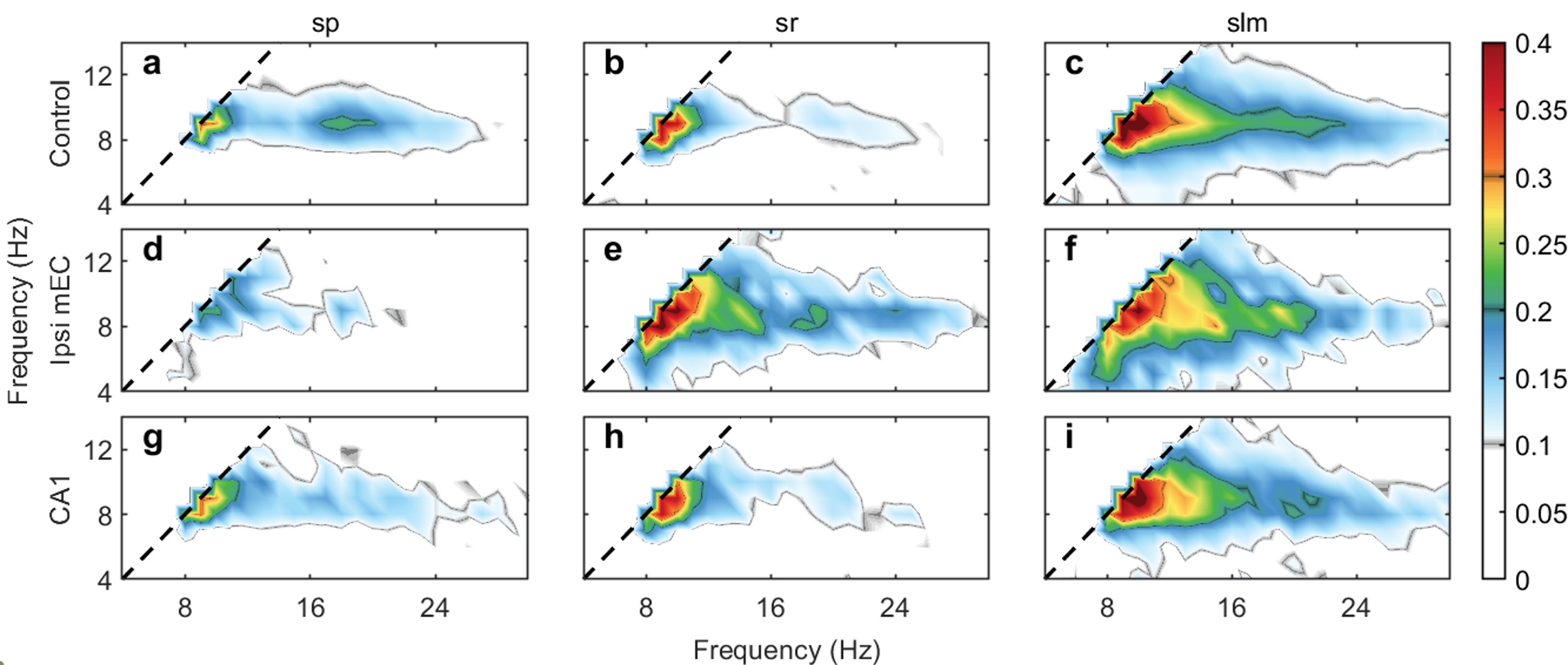


Supplemental 1: The data in this figure is the same as Figure 6. This figure is enlarged at low frequency ranges for clarity.

**
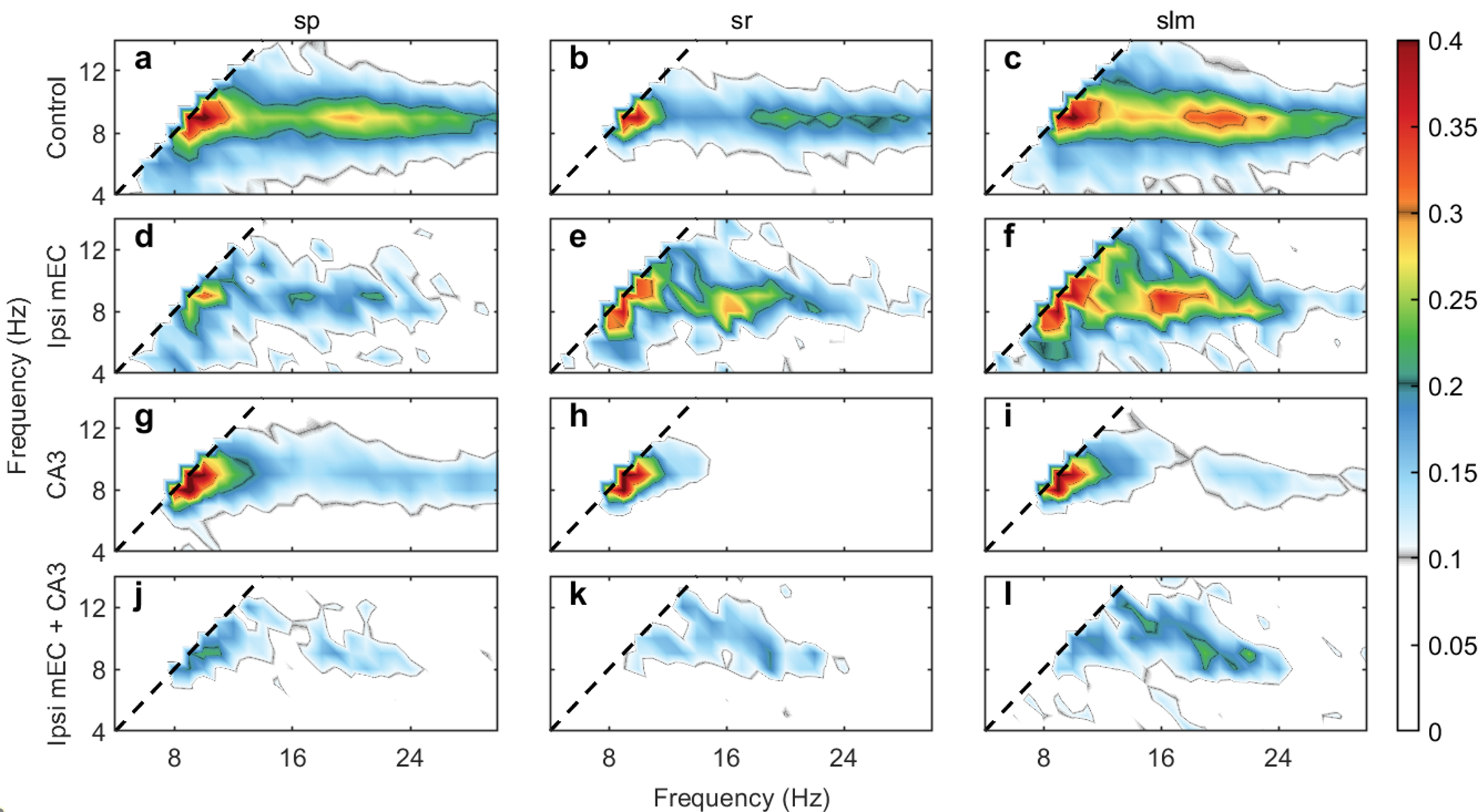
**

Supplemental 2: The data in this figure is the same as Figure 7. This figure is enlarged at low frequency ranges for clarity.

**
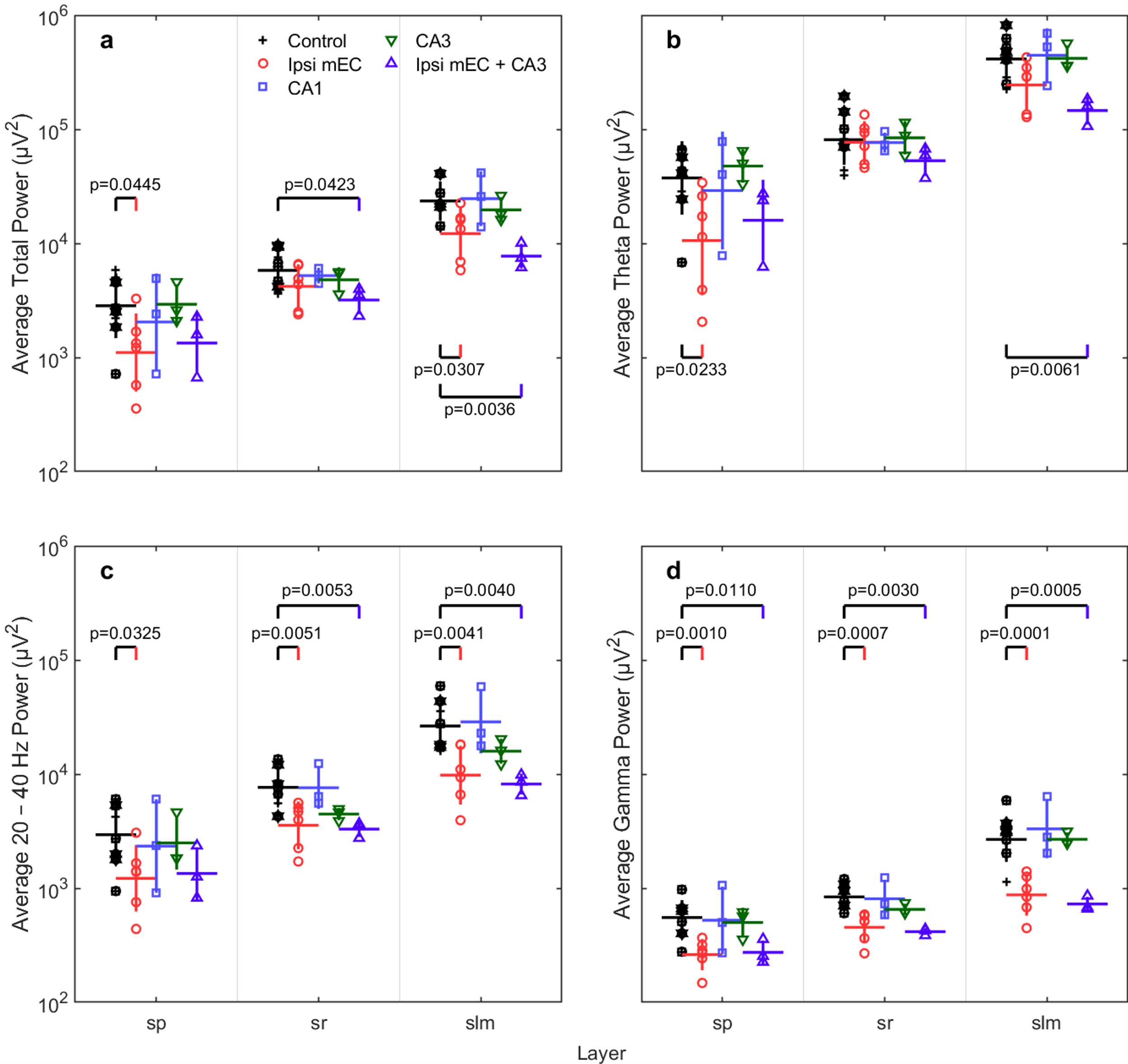
**

Supplemental 3: The data in Subfigure a-d is the same as Figure 3f-i. This figure is enlarged for better markers visualization.

**
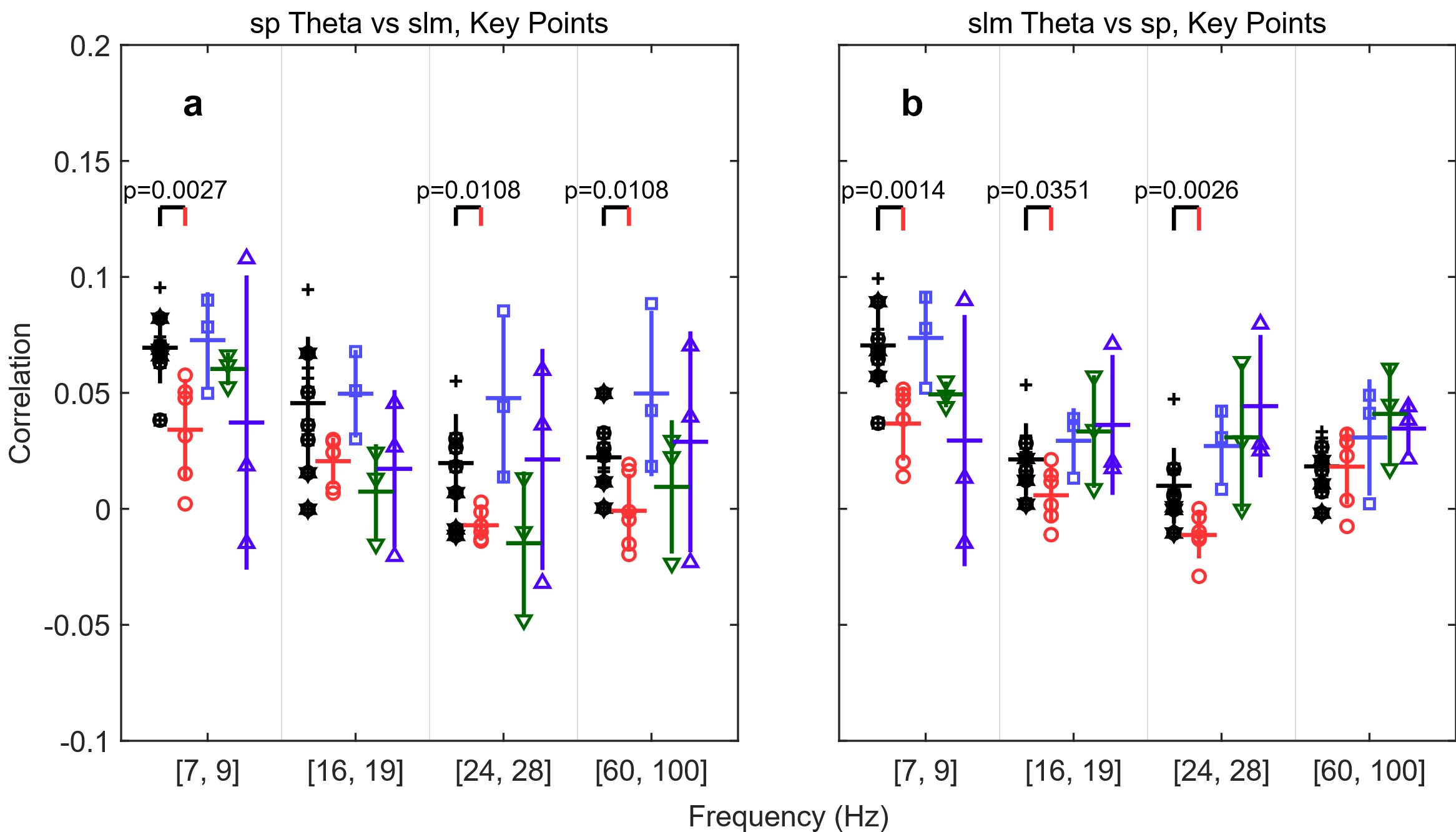
**

Supplemental 4: The data in Subfigure a and b is the same as Figure 4k and l. This figure is enlarged for better markers visualization.

**
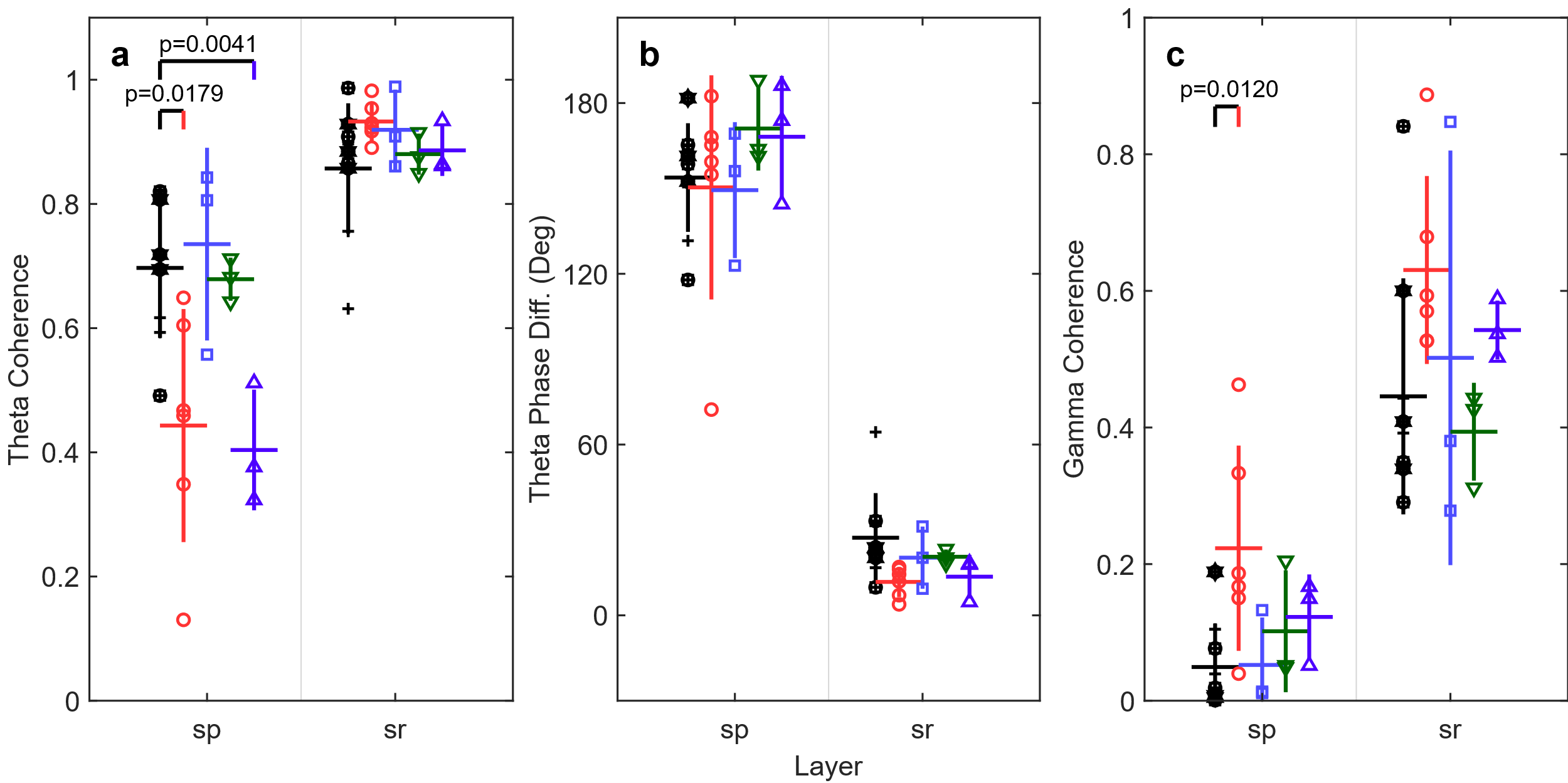
**

Supplemental 5: The data in Subfigure a-c is the same as Figure 5n-p. This figure is enlarged for better markers visualization.
